# Dilute Aqueous-Aprotic Electrolyte Towards Robust Zn-Ion Hybrid Supercapacitor with High Operation Voltage and Long Lifespan

**DOI:** 10.1007/s40820-024-01372-x

**Published:** 2024-03-25

**Authors:** Shuilin Wu, Yibing Yang, Mingzi Sun, Tian Zhang, Shaozhuan Huang, Daohong Zhang, Bolong Huang, Pengfei Wang, Wenjun Zhang

**Affiliations:** 1 Key Laboratory of Catalysis and Energy Materials Chemistry of Ministry of Education & Hubei Key Laboratory of Catalysis and Materials Science, South-Central Minzu University, Wuhan, 430074 People’s Republic of China; 2 Department of Materials Science and Engineering, and Center of Super-Diamond and Advanced Films, City University of Hong Kong, 83 Tat Chee Avenue, Kowloon Tong, Hong Kong SAR People’s Republic of China; 3 Department of Applied Biology and Chemical Technology, The Hong Kong Polytechnic University, Hung Hom, Kowloon, Hong Kong SAR People’s Republic of China; 4 Key Laboratory of Photochemical Conversion and Optoelectronic Materials, Technical Institute of Physics and Chemistry, Chinese Academy of Sciences, Beijing, People’s Republic of China

**Keywords:** Zn-ion supercapacitors, Zn metal anode, Electrolyte engineering, Hydrogen bonds, Solvation structures

## Abstract

With the merits of the high energy density of batteries and power density of supercapacitors, the aqueous Zn-ion hybrid supercapacitors emerge as a promising candidate for applications where both rapid energy delivery and moderate energy storage are required. However, the narrow electrochemical window of aqueous electrolytes induces severe side reactions on the Zn metal anode and shortens its lifespan. It also limits the operation voltage and energy density of the Zn-ion hybrid supercapacitors. Using ‘water in salt’ electrolytes can effectively broaden their electrochemical windows, but this is at the expense of high cost, low ionic conductivity, and narrow temperature compatibility, compromising the electrochemical performance of the Zn-ion hybrid supercapacitors. Thus, designing a new electrolyte to balance these factors towards high-performance Zn-ion hybrid supercapacitors is urgent and necessary. We developed a dilute water/acetonitrile electrolyte (0.5 m Zn(CF_3_SO_3_)_2_ + 1 m LiTFSI-H_2_O/AN) for Zn-ion hybrid supercapacitors, which simultaneously exhibited expanded electrochemical window, decent ionic conductivity, and broad temperature compatibility. In this electrolyte, the hydration shells and hydrogen bonds are significantly modulated by the acetonitrile and TFSI^−^ anions. As a result, a Zn-ion hybrid supercapacitor with such an electrolyte demonstrates a high operating voltage up to 2.2 V and long lifespan beyond 120,000 cycles.

**Supplementary Information:**

The online version contains supplementary material available at 10.1007/s40820-024-01372-x.

## Highlights

- A novel aqueous/aprotic electrolyte with low salt concentration (i.e., 0.5 m Zn(CF_3_SO_3_)_2_+1 m LiTFSI) demonstrated an expanded electrochemical window, which can simultaneously stabilize Zn metal anode and increase the operation voltage of Zn-ion hybrid supercapacitors.
- The coordination shell of the electrolyte induced by acetonitrile and LiTFSI can not only suppress the Zn corrosion and hydrogen evolution reaction but also promote the cathodic stability and ion migration, which is depicted by the density functional theory simulations together with experimental characterizations.
- The Zn-ion hybrid supercapacitor based on the developed electrolyte can operate within 0–2.2 V in a wide temperature range with an ultra-long lifespan (> 120,000 cycles).

**Supplementary Information:**

The online version contains supplementary material available at 10.1007/s40820-024-01372-x.

## Graphical abstract

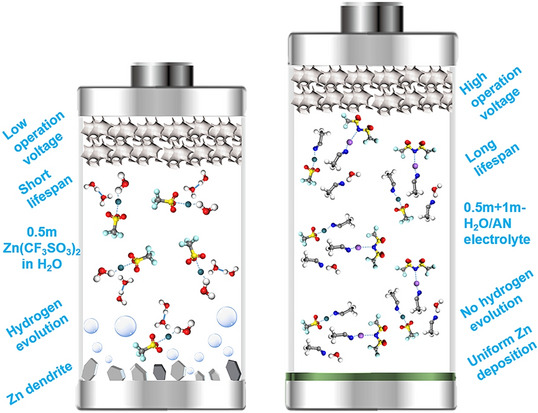

## Introduction

With the merits of high-power output and long lifespan, supercapacitors bridge the gap between traditional capacitors and rechargeable batteries, and therefore have been widely used for diverse commercial applications, such as hybrid electrical vehicles, grid power buffer, and energy harvesting systems [1, 2]. Compared with rechargeable batteries, supercapacitors can accept and deliver energy much more rapidly, and tolerate magnitude more charge–discharge cycles. However, the relatively low energy density of the supercapacitors is the bottleneck of their applications. Building a hybrid supercapacitor that consists of a battery-type anode for an energy reservoir and a capacitive-type carbonaceous cathode for the power supplier can improve the energy density of supercapacitors and maintain their power output. In the different configurations of hybrid supercapacitors, metallic Zn is an attractive anode material due to its high specific capacity (820 mAh g^−1^ or 5,855 mAh cm^−3^), low redox potential (− 0.76 V vs. standard hydrogen electrode), low cost, and natural abundance [3–8]. With the particular effort in engineering the carbonaceous cathode materials to obtain a high specific capacitance, e.g., N-doped carbon with hierarchical porosity, pencil-derived carbon, B/N co-doped carbon, and metal–organic frame derived carbon, a significantly improved energy density than those of other conventional supercapacitors has been demonstrated thus far (~ 100 Wh kg^−1^) [7, 9–15].

Albeit significant progress has been achieved in developing novel carbonaceous materials for aqueous Zn-ion hybrid supercapacitors, problems induced by the narrow electrochemical window of aqueous electrolytes remain to be solved. For example, the narrow electrochemical window of aqueous electrolytes induces severe side reactions (e.g., hydrogen evolution and galvanic corrosion) on the Zn metal anode, shortening the lifespan of the Zn-ion hybrid supercapacitors to be far behind those of electrochemical double-layer capacitors (usually > 100,000 cycles) [16–24]. Moreover, the aqueous electrolytes (e.g., ZnSO_4_) can only enable the Zn-ion hybrid supercapacitors operating in a finite operation voltage range (typically 0.2–1.8 V) because of the decomposition of water, resulting in an unsatisfactory energy density [4, 10, 25–30].

Electrolyte engineering has demonstrated its vital role in improving the energy density and lifespan of aqueous energy storage devices such as batteries and supercapacitors, and this approach is especially attractive in the aspect of practical applications since it can be easily scaled up. For example, through the strategy of “water in salt” by dissolving a high concentration of salts in water, the electrochemical window of aqueous electrolytes can be effectively expanded. In such electrolytes, most of the water molecules are solvated with the cations and/or anions, the decomposition of water molecules is thus suppressed, and the stability of Zn anode is promoted due to the reduced irreversible reactions between Zn and electrolytes [3, 7, 31–33]. Moreover, many electrolyte additives, such as surfactants (e.g., sodium dodecyl sulfate (SDS)) [34], polymers (e.g., PEG) [35], and small organic molecules (e.g., urea, sugar, methanol, glucose, succinonitrile, and NMP) [33, 36–39], have also been shown to be able to enhance the stability of Zn metal anode in aqueous Zn-ion batteries, either by forming an adsorption layer on the surface of Zn anode or altering the solvation structures of electrolytes to suppress the corrosion of Zn anode and hydrogen evolution. However, the use of super-concentrated salts or other additives inevitably increases the electrolyte density and viscosity, which leads to decreased energy density at device level and sacrifices of rate capability of devices [40]. In addition, the cost of the electrolyte, especially in the cases of using high-cost fluorine-based electrolytes is another concern [41, 42].

In this work, we develop an aqueous-aprotic electrolyte with low salt concentrations, i.e., 0.5 m Zn(CF_3_SO_3_)_2_ and 1 m LiTFSI in a mixture of water and acetonitrile, and demonstrate that the electrolyte has a broad electrochemical window and decent ionic conductivity, which is favorable to promoting the electrochemical performance of Zn-ion hybrid supercapacitors (i.e., the operation voltage and the lifespan). Theoretical calculations verify that acetonitrile molecules can competitively replace the water molecules to form primary solvation structures with Zn^2+^ cations, and break the hydrogen bonds among water molecules to reduce their chemical activity. As a result, the hydrogen evolution reaction and galvanic corrosion are remarkably suppressed on the Zn metal anode to prolong its lifetime. Most importantly, a wide operating voltage range of 0–2.2 V has been reached for the first time in Zn-ion hybrid supercapacitors with excellent cyclic stability up to 120,000 cycles. In addition, the electrolyte also enables the Zn-ion hybrid supercapacitors to operate at temperatures in a wide temperature range as low as − 30 °C. This work provides a new approach for designing electrolytes for high-performance Zn-ion hybrid supercapacitors, which is also applicable for other aqueous energy storage devices such as Zn-ion batteries.

## Experimental Section

### Preparation of the Water-Acetonitrile Electrolytes

A certain amount of lithium bis-trifluoromethanesulfonimide (LiTFSI, J&K^®^) was first dissolved in deionized water to obtain a 21 m LiTFSI aqueous electrolyte, where *m* means molar salts dissolved in per kilogram solvent. Then, the corresponding mass of anhydrous acetonitrile (J&K^®^) was added to dilute the 21 m LiTFSI aqueous electrolyte into certain concentrations (e.g., 1, 3, and 5 m). Finally, the zinc trifluoromethanesulfonate (Zn(CF_3_SO_3_)_2_, J&K^®^) was added into the diluted electrolyte to reach 1 m based on the total mass of water and acetonitrile, and the water-acetonitrile electrolytes were obtained.

### Synthesis of aMEGO

The aMEGO powder was synthesized following the steps reported previously [43]. Briefly, graphite oxide (GO) powder obtained by modified Hummers’ method was reduced in a microwave oven with a power of 800 W for 1 min, to prepare the microwave-exfoliated graphene oxide (MEGO) powder. Then, the MEGO powder was mixed with KOH aqueous solution and heated to evaporate water to get a uniform KOH/MEGO mixture with a mass ratio of 7:1. The mixture was further annealed in Ar at 800 °C for 2 h and naturally cooled down to room temperature. After sequentially washing with dilute HCl solution, deionized water, and ethanol and dried in the oven at 60 °C for 6 h, the aMEGO powder was obtained.

### Characterization

The morphology of electrodes before and after cycling was investigated by a field-emission scanning electron microscope (Philips, FEG-XL30) with an accelerating voltage of 5 kV. The structure analysis was performed by X-ray diffraction (XRD) on Bruker D2 system with Cu K_α_ irradiation (λ = 1.5418 Å, tube voltage: 30 kV and tube current: 10 mA). The Raman spectra of electrolytes were taken on a Renishaw^®^ inVia system with a 514 nm laser and 5 × objective. The ^1^H nuclear magnetic resonance (NMR) spectra were collected on a Bruker Advance III 400 NMR system, and a co-axial glass tube was used to conduct the experiments with CDCl_3_ (99.8 atom% D, contains 0.03% v/v TMS; J&K) as magnetic field locker. The chemical shifts were calibrated with the peak of tetramethylsilane in CDCl_3_ as 0 ppm.

### Electrochemical Test

The Tafel plots were measured based on a three-electrode system with a scan rate of 10 mV s^−1^, in which the Pt foil, Zn foil, and Ag/AgCl electrode were used as the counter electrode, working electrode, and reference electrode, respectively. The galvanic corrosion of the Zn foil was evaluated based on a Zn-Ti galvanic cell in a CR2032 coin cell. The Coulombic efficiency of Zn stripping/plating was measured based on a CR2032 coin cell, in which Zn foil (50 μm), Cu foil (20 μm) and glass fiber (Whatman^®^) were used as anode, cathode and separator, respectively. The cell was initially discharged for a certain time with a specific current density and then charged to 0.5 V at the same current density for each cycle on a Neware^®^ battery testing system. To prepare cathode electrode for the evaluation of Zn-ion hybrid supercapacitors, aMEGO powder was added into polytetrafluoroethylene (PTFE, 60 wt% aqueous dispersion, commercial purchased from Aladdin^®^) slurry binder at a mass ratio of 5:95, then rolled into a thin film with a thickness around 60–70 μm. Then, the film was punched into disks with a diameter of 10 mm, pressed into a Ti mesh (14 mm diameter) by hydraulic press at 20 MPa for 30 s and dried in a vacuum oven at 100 °C for 6 h. The hybrid supercapacitors were assembled into CR2032 coin-type cells with 16 mm Zn foil, aMEGO and glass fiber (Whatman^®^) as anode, cathode and separator, respectively. The electrochemical performance was evaluated by cyclic voltammetry and galvanostatic charge–discharge techniques on an electrochemical workstation and Neware^®^ battery testing system, respectively. The specific gravimetric capacitance was calculated based on the equation: *C* = *It/mU*, where *I* is the charge/discharge current, *t* is the discharge time, *m* is the mass of the aMEGO, and *U* the is voltage difference of the discharge curve. The energy density was calculated using the equation: *E* = *1/2CV*^*2*^, where *C* is the specific gravimetric capacitance calculated above, and *V* is the operation voltage of the hybrid supercapacitors. The power density was calculated by the equation: *P* = *E/t*, where *E* is the energy density and *t* is the corresponding discharge time [44]. The electrochemical impedance spectroscopy was conducted on an electrochemical workstation with a frequency range from 100 kHz to 100 mHz and an amplitude of 10 mV.

### Calculation Setup

To investigate the electronic structures of different electrolytes, density functional theory (DFT) within the CASTEP package has been applied for all calculations in this work. We have chosen the generalized gradient approximation (GGA) with Perdew-Burke-Ernzerhof (PBE) functions for the descriptions of exchange–correlation interactions. The plane-wave cutoff energy has been set to 380 eV based on ultrafine quality and the ultrasoft pseudopotential scheme. The coarse k-point has been applied for the energy minimization based on the Broyden–Fletcher–Goldfarb–Shannon (BFGS) algorithm. The Hellmann–Feynman forces should be converged to less than 0.001 eV Å^−1^ and the total energy should be less than 5 × 10^–5^ eV atom^−1^. The convergence criteria maximum stress and atomic displacement have been set to 0.2 GPa and 0.005 Å, respectively.

The molecular dynamic (MD) simulations have been conducted in four electrolytes including 0.5 m + 1 m-H_2_O electrolyte, 0.5 m + 1 m-H_2_O/AN electrolyte, 0.5 m + 3 m-H_2_O/AN electrolyte, and 0.5 m + 5 m-H_2_O/AN electrolyte. The molar ratio of these three electrolyte models are Zn (CF_3_SO_3_)_2_: LiTFSI: H_2_O = 0.5: 1: 55.6; Zn (CF_3_SO_3_)_2_: LiTFSI: H_2_O: AN = 0.5: 1: 2.6: 23.2; Zn(CF_3_SO_3_)_2_: LiTFSI: H_2_O: AN = 0.5: 3: 7.9: 20.9; and Zn(CF_3_SO_3_)_2_: LiTFSI: H_2_O: AN = 0.5: 5: 13.2: 18.6, respectively. The MD has been carried out under the NVT condition in 298 K. The time step is 1 fs and the total simulation time has been set to 3 ps. We have selected the Noise scheme for the thermostat. After the MD simulations, we further carry out geometry optimizations to investigate the electronic structures and the interaction energies.

## Results and Discussion

### Electrochemical Performance of the Zn Metal Anode in the Zn(CF_3_SO_3_)_2_ + LiTFSI Water/Acetonitrile Electrolytes

**Fig. 1:**
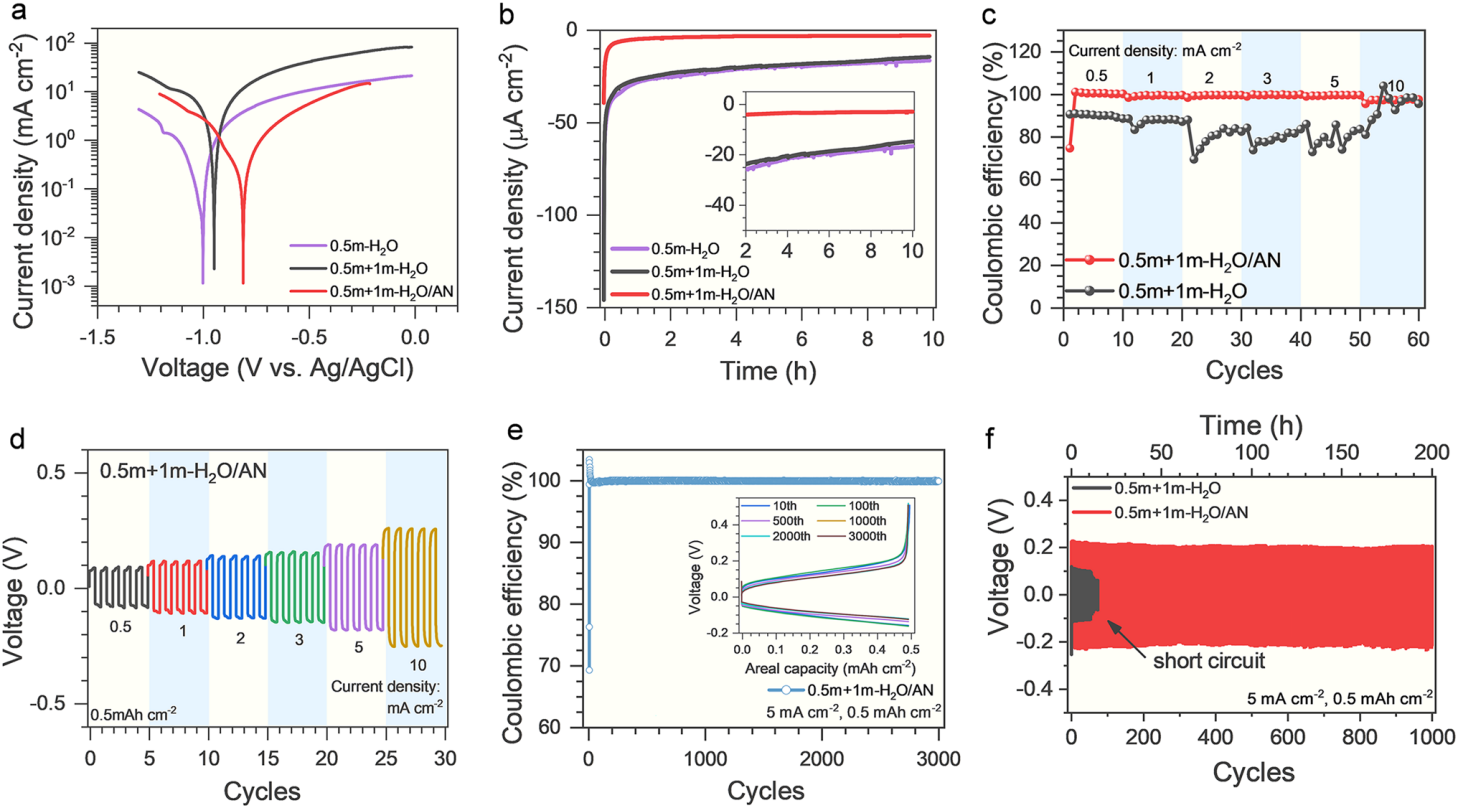
Evaluation of the stability of Zn metal anode in 0.5 m Zn(CF_3_SO_3_)_2_ + 1 m LiTFSI H_2_O/AN electrolyte. **a** Tafel plots of Zn plating/stripping in the 0.5 m + 1 m-H_2_O/AN and reference aqueous electrolytes. **b** The plots of current density versus time of the Zn-Ti galvanic cells in the 0.5 m + 1 m-H_2_O/AN and reference aqueous electrolytes. **c** Coulombic efficiencies of the Zn-Cu cells with the 0.5 m + 1 m-H_2_O/AN and reference aqueous electrolytes at various current densities (0.5–10 mA cm^−2^). **d** Voltage profiles of Zn-Zn symmetric cells in the 0.5 m + 1 m-H_2_O/AN at various current densities (0.5–10 mA cm^−2^). **e** Coulombic efficiency of the Zn-Cu cell with the 0.5 m + 1 m-H_2_O/AN electrolyte at 5 mA cm^−2^, inset is the charge–discharge curves at specific cycles (e.g., the 10th, 100th, 500th, 1000th, 2000th and 3000th cycle). **f** Voltage profiles of the Zn-Zn symmetric cells with the 0.5 m + 1 m-H_2_O/AN and reference aqueous electrolytes at 5 mA cm^−2^; the areal capacity of these tests is 0.5 mAh cm^−2^ for each cycle

The electrochemical performance of Zn metal anodes was evaluated in the water/acetonitrile (H_2_O/AN) electrolytes, and the 0.5 m Zn(CF_3_SO_3_)_2_ aqueous electrolyte (denoted as 0.5 m-H_2_O) and 0.5 m Zn(CF_3_SO_3_)_2_ + 1 m LiTFSI aqueous electrolyte (denoted as 0.5 m + 1 m-H_2_O) as references. From the Tafel plots of Zn metal in the H_2_O/AN electrolytes (Figs. 1a and S1), a sharp peak was observed at potentials of -0.77, -0.82, and -0.83 V (vs. Ag/AgCl electrode) in the 0.5 m + 1 m-H_2_O/AN, 0.5 m + 3 m-H_2_O/AN, and 0.5 m + 5 m-H_2_O/AN electrolytes, respectively. By contrast, the peaks at potentials of − 0.99 and − 0.94 V were detected in the 0.5 m-H_2_O and 0.5 m + 1 m-H_2_O electrolytes, respectively. In general, the peak potential represents the initial potential of Zn plating/stripping processes, and a higher potential is favorable to avoid the hydrogen evolution which is a competitive process accompanied by Zn plating [18, 45]. Thus, the higher potentials of Zn plating/stripping processes in the H_2_O/AN electrolytes indicate an effectively suppressed hydrogen evolution reaction (HER), which is favorable for improving the Coulombic efficiency of Zn plating/stripping.

Moreover, a galvanic cell was built to investigate the thermodynamic stability of Zn metal anode in the H_2_O/AN electrolytes. From the time-dependent corrosion current curves (Figs. 1b and S2), the current densities of the galvanic cells are initially large (~ 40 μA cm^−2^) which are partially ascribed to the capacitive contribution. Then the current densities are quickly dropped by one order of magnitude to µA cm^−2^ level. Specifically, the current densities of the galvanic cells are stabilized at 3–5 µA cm^−2^ in the H_2_O/AN electrolytes. By contrast, the current densities of the galvanic cells fluctuate around 20 µA cm^−2^ in the reference electrolytes. The much reduced corrosion currents imply that the galvanic corrosion is drastically reduced in the H_2_O/AN electrolytes. Correspondingly, the SEM images (Fig. S3) of the Zn foils after galvanic corrosion maintain a smooth surface in the H_2_O/AN electrolytes, whereas severely corroded surfaces are observed in the reference electrolytes. Moreover, the SEM images (Fig. S4) and XRD patterns (Fig. S5) of the Zn foils after soaking in the H_2_O/AN and reference electrolytes also demonstrate the anti-corrosion capability of the H_2_O/AN electrolytes. These results together reveal that the Zn metal is thermodynamically more stable in the H_2_O/AN electrolytes.

The Coulombic efficiency of Zn plating/stripping was further tested based on a Zn-Cu coin cell with the H_2_O/AN electrolytes. As shown in Fig. S6, the cells with H_2_O/AN electrolytes show stable and significantly higher Coulombic efficiency (average > 99%) after 100 cycles as expected, while the cells with aqueous electrolyte (0.5 m + 1 m-H_2_O) demonstrate gradually decreasing and fluctuating Coulombic efficiency (from 90 to 70% after 50 cycles) under the same condition. Moreover, for the H_2_O/AN electrolytes with different concentrations of LiTFSI (1–5 m), the initial Coulombic efficiency decreases with the increase of LiTFSI concentration, and the average Coulombic efficiency of Zn plating/stripping is the highest in 0.5 m + 1 m-H_2_O/AN electrolyte. From the SEM images of Zn foils in the Zn-Cu cells after specific cycles (Fig. S7), a great number of macro-pores are observed on the Zn foils after the 10th, 20th, and 50th cycles in the aqueous electrolytes (0.5 m-H_2_O and 0.5 m + 1 m-H_2_O), which indicates an uneven Zn plating/stripping behavior and severe Zn corrosion during cycling. Nevertheless, a compact and uniform surface is revealed on the Zn foils cycled in 0.5 m + 1 m-H_2_O/AN electrolyte, confirming an excellent Zn plating/stripping reversibility in 0.5 m + 1 m-H_2_O/AN electrolyte. At enlarged current densities up to 10 mA cm^−2^, a high Coulombic efficiency (97.3% on average) is still maintained in 0.5 m + 1 m-H_2_O/AN electrolyte, in contrast to its rapid decrease and fluctuation in the aqueous electrolyte (0.5 m + 1 m-H_2_O), as shown in Figs. 1c and S8. However, for the H_2_O/AN electrolytes with further increased LiTFSI concentrations (0.5 m + 3 m-H_2_O/AN and 0.5 m + 5 m-H_2_O/AN), a decreased Coulombic efficiency and increased voltage hysteresis are observed at higher current densities (e.g., > 5 mA cm^−2^, Fig. S9), indicating that an excessive concentration of LiTFSI is unfavorable to the reversibility of Zn plating/stripping at high current densities. Correspondingly, the symmetric Zn-Zn cells with 0.5 m + 1 m-H_2_O/AN electrolyte only show slightly increased voltage hysteresis with enlarged current densities (Fig. 1d), while a dramatically increased voltage hysteresis followed by short-circuit is observed for 0.5 m + 3 m-H_2_O/AN and 0.5 m + 5 m-H_2_O/AN electrolytes (Fig. S10). After cycling at enlarged current densities (e.g., 5 mA cm^−2^), the Zn foil maintains uniform and compact morphology in 0.5 m + 1 m-H_2_O/AN electrolyte, in accordance with a low voltage hysteresis even at high current densities. In contrast, the Zn foils cycled in 0.5 m + 3 m-H_2_O/AN and 0.5 m + 5 m-H_2_O/AN electrolytes show a rough surface, revealing an uneven plating behavior at increased current densities (Fig. S11). These observations demonstrate that excessive LiTFSI in electrolytes is unfavorable to the transportation of Zn^2+^ ions and deteriorates the rate performance of Zn metal anode, which is in agreement with the larger charge transfer resistances in the Zn-Zn symmetric cells with increased LiTFSI concentrations (Fig. S12). The long-term cyclic stability of the Zn metal anode in the 0.5 m + 1 m-H_2_O/AN electrolyte was further verified by the Zn-Cu and Zn-Zn cells. A high average Coulombic efficiency of 97.3% is achieved in the 0.5 m + 1 m-H_2_O/AN electrolyte for more than 3,000 cycles (Fig. 1e), and no obvious changes are observed in the charge–discharge curves of specific cycles (the inset of Fig. 1e). Correspondingly, the Zn-Zn cells with the 0.5 m + 1 m-H_2_O/AN electrolyte also show stable voltage hysteresis with prolonged cycles, while a short-circuit quickly occurs in the Zn-Zn cells with the reference electrolyte (0.5 m + 1 m-H_2_O), as shown in Fig. 1f. These results further verify the superior stability of the Zn metal anode in the 0.5 m + 1 m-H_2_O/AN electrolyte.

### Electrochemical Performance of the Zn-ion Hybrid Supercapacitors in the Zn(CF_3_SO_3_)_2_ + LiTFSI Water/Acetonitrile Electrolytes

**Fig. 2:**
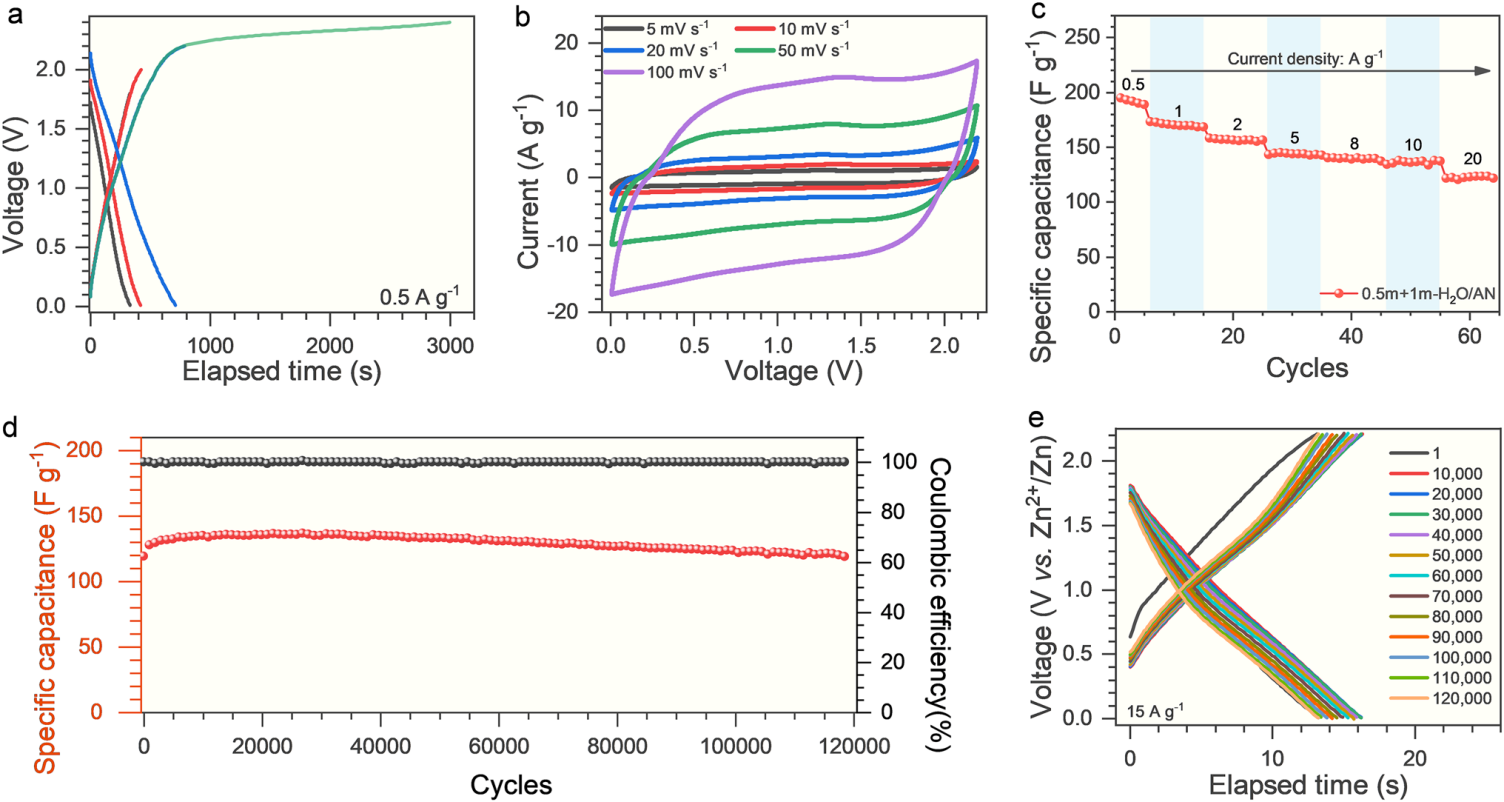
Electrochemical performance of the Zn-aMEGO hybrid supercapacitor with 0.5 m + 1 m-H_2_O/AN electrolyte. **a** Galvanostatic charge–discharge curves of the device with different voltage ranges at 0.5 A g^−1^. **b** Cyclic voltammetry curves of the device at various scan rates (5–100 mV s^−1^) within 0–2.2 V. **c** Rate capability of the device within a voltage range of 0–2.2 V. **d** Cycling stability of the device at 15 A g^−1^ within 0–2.2 V. **e** The galvanostatic charge–discharge curves of the device after specific cycles at 15 A g^−1^

To assess the practical applicability of H_2_O/AN electrolytes, a prototype of a Zn-ion hybrid supercapacitor was fabricated, in which porous graphene (aMEGO) with high specific capacitance and excellent cyclic stability was used as the cathode material [46], and Zn foil and glass fiber were used as anode and separator, respectively. First, the operation voltages of the hybrid supercapacitors were assessed in 0.5 m + 1 m-H_2_O/AN electrolyte by the galvanostatic charge–discharge and cyclic voltammetry techniques. As shown in Fig. 2a, the charge–discharge curves of the Zn-aMEGO hybrid supercapacitors maintain linear and symmetric within the voltage up to 2.2 V, and a plateau is observed when the voltage is above 2.2 V. Correspondingly, the cyclic curves (Fig. S13) exhibit a quasi-rectangular shape in the voltage range of 0–2.2 V, and have an obvious current leap at voltages beyond 2.2 V. These results suggest an optimal electrochemical window of 0–2.2 V. In sharp contrast, only a narrow operation voltage range of 0.2–1.8 V can be achieved in the reference electrolyte (0.5 m + 1 m-H_2_O) under the same test condition (Fig. S14). In addition, the linear scan voltammetry curves of the Zn-Ti cells also verify the superior cathodic stability of 0.5 m + 1 m-H_2_O/AN electrolyte (Fig. S15).

Furthermore, the rate performance of the Zn-aMEGO hybrid supercapacitors with 0.5 m + 1 m-H_2_O/AN electrolyte was evaluated within the voltage range of 0–2.2 V. The cyclic voltammetry curves (Fig. 2b) remain a quasi-rectangular shape with little distortion as the scan rates increase from 5 to 100 mV s^−1^, and a high capacitance retention up to 64% is also achieved as the current density increases by 40 times from 0.5 to 20 A g^−1^ (Figs. 2c and S16). By contrast, the cyclic voltammetry curves become obviously distorted with decreasing integral areas at the enlarged scan rates in 0.5 m + 3 m-H_2_O/AN and 0.5 m + 5 m-H_2_O/AN electrolytes (Fig. S17), which indicates an inferior rate performance. These results demonstrate that the Zn-aMEGO hybrid supercapacitors have an excellent rate performance in 0.5 m + 1 m-H_2_O/AN electrolyte as summarized in a Ragone plot (Fig. S18), benefiting from its high ionic conductivity (23 mS cm^−1^) and small charge transfer resistance (Fig. S19). The cyclic stability of the Zn-aMEGO hybrid supercapacitors with 0.5 m + 1 m-H_2_O/AN electrolyte was further tested, and a high capacitance retention up to 88% was maintained even after 120,000 cycles with a high operation voltage range of 0–2.2 V (Fig. 2d, e). A clear and smooth surface of the aMEGO electrode was observed from the SEM images (Fig. S20a, b), and no byproducts were detected from the XRD diffraction pattern (Fig. S20c). These results together verify that the 0.5 m + 1 m-H_2_O/AN electrolyte has superior electrochemical stability and as well the capability to stabilize Zn metal anodes.

**Fig. 3:**
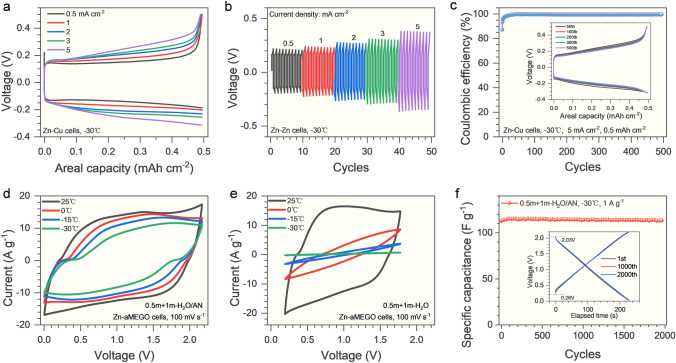
The electrochemical performance of 0.5 m + 1 m-H_2_O/AN electrolyte at low temperature (− 30 °C). **a** Charge–discharge curves of the Zn-Cu cells at different current densities. **b** Voltage profiles of the Zn-Zn cells at various current densities. **c** Coulombic efficiency of the Zn-Cu cells at 5 mA cm^−2^, inset is the charge–discharge curves at the specific cycles. The cyclic voltammetry curves of the Zn-aMEGO hybrid supercapacitors with **d** the 0.5 m + 1 m-H_2_O/AN electrolyte and **e** the 0.5 m + 1 m-H_2_O aqueous electrolyte under different temperatures at a scan rate of 100 mV s^−1^. **f** The capacitance retention of the Zn-aMEGO supercapacitors with 0.5 m + 1 m-H_2_O/AN electrolyte at 1 A g^−1^ within a voltage range of 0–2.2 V, inset is the charge–discharge curves of the device after specific cycles

The low temperature tolerance of the H_2_O/AN electrolytes was also investigated to assess their applicability under harsh conditions. From Fig. 3a, in 0.5 m + 1 m-H_2_O/AN electrolyte, a high Coulombic efficiency and slightly increased voltage hysteresis are observed with ever-increasing current densities based on the Zn-Cu cells at a reduced temperature (− 30 °C). Consistently, the Zn-Zn symmetric cells also demonstrate a slightly increased yet stable voltage hysteresis at enlarged current densities (Fig. 3b). Moreover, a high average Coulombic efficiency (99.4%) and excellent cyclic stability (over 500 cycles) are achieved in 0.5 m + 1 m-H_2_O/AN electrolyte at − 30 °C based on the Zn-Cu cells (Fig. 3c). These observations indicate that a high reversibility and rapid kinetics of Zn plating/stripping are retained in 0.5 m + 1 m-H_2_O/AN electrolyte even at a harsh temperature condition. As a result, the Zn-aMEGO supercapacitors demonstrate an excellent rate performance within a wide temperature range in the 0.5 m + 1 m-H_2_O/AN electrolyte, as verified by the little distortion and slightly decreased integral areas of cyclic voltammetry curves (Fig. 3d). In contrast, in the 0.5 m + 1 m-H_2_O electrolyte, the cyclic voltammetry curves become distorted with drastically decreased integral areas at lower testing temperatures (Fig. 3e). The Zn-aMEGO supercapacitors with 0.5 m + 1 m-H_2_O/AN electrolyte could stably operate under − 30 °C for more than 2,000 cycles (Fig. 3f). As the concentration of LiTFSI increases, however, inferior tolerance to the low temperatures with obviously distorted cyclic voltammetry curves were observed for 0.5 m + 3 m-H_2_O/AN and 0.5 m + 5 m-H_2_O/AN electrolytes (Fig. S21). The excellent low temperature performance of the 0.5 m + 1 m-H_2_O/AN electrolyte is believed to be associated with its high ionic conductivities at low temperatures (7 mS cm^−1^ at − 30 °C, Fig. S22).

### Solvation Structures of the Zn(CF_3_SO_3_)_2_ + LiTFSI Water/Acetonitrile Electrolytes

To understand the electrolyte performances, the solvation structures of H_2_O/AN electrolytes were comprehensively studied. From the Raman spectra (Fig. S23), as compared with reference electrolytes (0.5 m-H_2_O and 0.5 m + 1 m-H_2_O), the intensity of the broad peak (3,100–3,700 cm^−1^) ascribed to hydrogen bonds among water molecules in the H_2_O/AN electrolytes is remarkably decreased, implying that most of the water molecules are solvated. Correspondingly, the Fourier Transform Infrared (FT-IR) spectra (Fig. S24) reveal valuable insights into the molecular interactions in the electrolytes. It is observed that the introduction of acetonitrile significantly reduces the intensity and width of the absorption peaks (2,800–3,700 cm^−1^) associated with the stretching vibrations of –OH groups in water. This suggests that acetonitrile disrupts the hydrogen bonding among water molecules. Furthermore, nuclear magnetic resonance (NMR) results (Fig. S25) demonstrate that the chemical shifts of the ^1^H in water shift to higher fields, indicating an increase in electron density as the concentration of LiTFSI increases. These results suggest that both acetonitrile and LiTFSI exhibit strong interactions with water molecules, thereby expanding the electrochemical window of water.

**Fig. 4:**
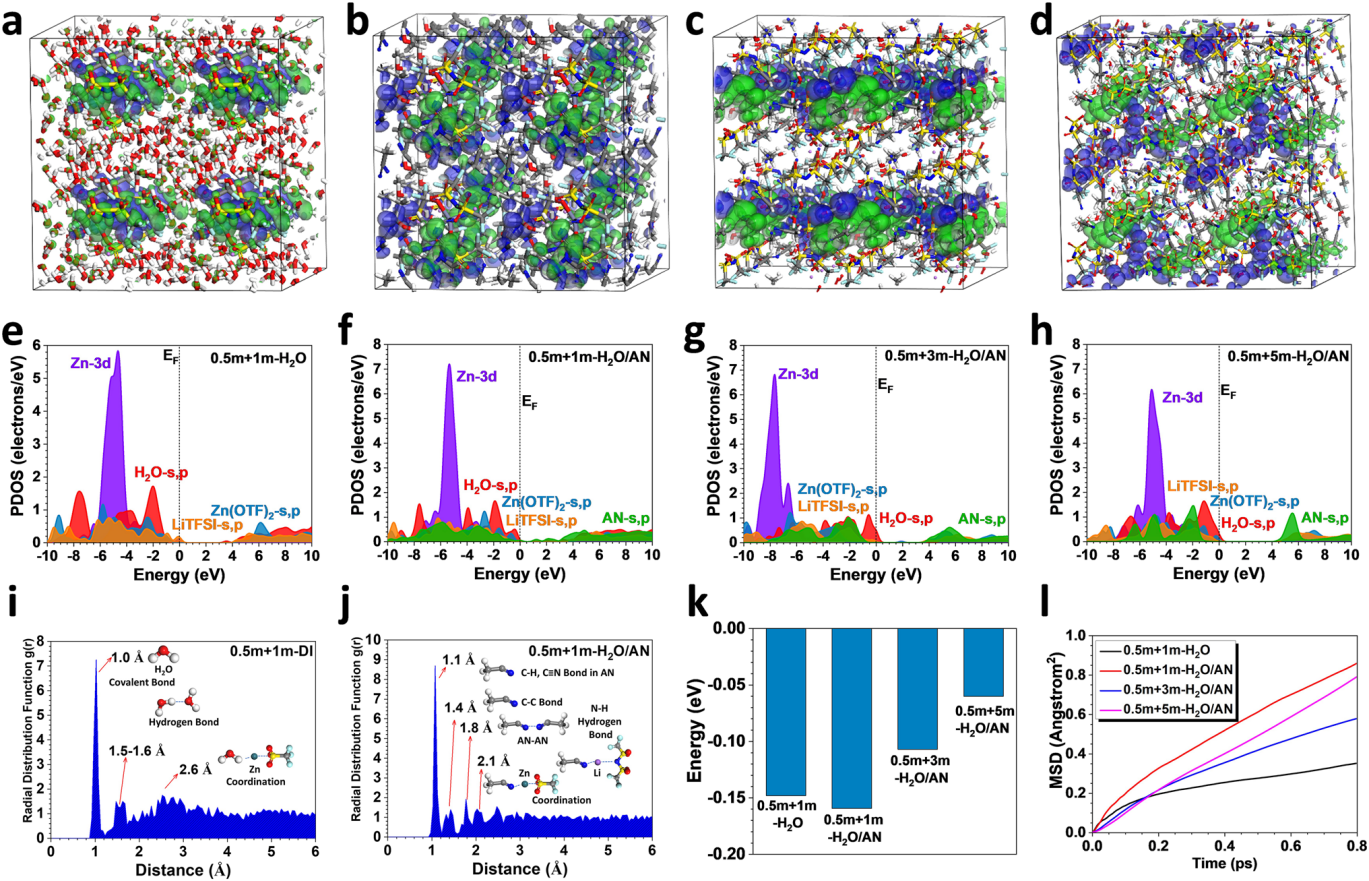
3D contour plots of the anti-bonding and bonding orbitals of **a** 0.5 m + 1 m-H_2_O electrolyte, **b** 0.5 m + 1 m-H_2_O/AN electrolyte, **c** 0.5 m + 3 m-H_2_O/AN electrolyte, and **d** 0.5 m + 5 m-H_2_O/AN electrolyte. Red balls = O, white balls = H, purple balls = Li, grey balls = Zn, yellow balls = S, dark blue balls = N, light blue balls = F; blue isosurface = bonding orbitals, green isosurface = anti-bonding orbitals. The PDOSs of **e** 0.5 m + 1 m-H_2_O electrolyte, **f** 0.5 m + 1 m-H_2_O/AN electrolyte, **g** 0.5 m + 3 m-H_2_O/AN electrolyte, and **h** 0.5 m + 5 m-H_2_O/AN electrolyte. The radial distribution function (RDF) comparison of **i** 0.5 m + 1 m-H_2_O electrolyte and **j** 0.5 m + 1 m-H_2_O/AN electrolyte. **k** Interaction energies comparison. **l** Mean square displacement (MSD) of different electrolytes at 298 K. (Color figure online)

We also carried out density functional theory (DFT) calculations of H_2_O/AN electrolytes with different concentrations through the molecular dynamic (MD) simulations. For the 0.5 m + 1 m-H_2_O electrolyte, the bonding orbitals and anti-bonding orbitals near the Fermi level are mostly near the Zn(CF_3_SO_3_)_2_ and TFSI^−^, respectively, where water molecules only limited contributed to the anti-bonding orbitals (Fig. 4a). The relatively weak orbital couplings between Zn(CF_3_SO_3_)_2_ and water molecules indicates that original solvation structure is not stable, which potentially leads to the side reactions and lower the overall performances. As the acetonitrile molecules are introduced in the electrolyte, the *s, p* orbitals of acetonitrile have strong contributions to both bonding and anti-bonding orbitals, especially near the C≡N bonds, implying that the acetonitrile dominates the interactions with both Zn(CF_3_SO_3_)_2_ and LiTFSI in 0.5 m + 1 m-H_2_O/AN, which is accompanied with improved orbital couplings (Fig. 4b). However, as the concentration of LiTFSI increases to 3 m, the bonding and anti-bonding orbitals are more dominated by LiTFSI, while the contributions of water and acetonitrile are alleviated (Fig. 4c). In this case, the energy barrier for electron transfer is increased. With the further increase of LiTFSI concentration to 5 m, the contributions of water molecules become much stronger in the bonding orbitals, which increases the possibility of HER (Fig. 4d). Meanwhile, the further weakened orbital couplings result in increased barriers for electron transfer, which is consistent with the enlarged resistance of experimental characterizations induced by the excessive LiTFSI.

Detailed electronic structures are revealed by the projected partial density of states (PDOSs). In 0.5 m + 1 m-H_2_O electrolyte, the Zn-3*d* orbitals show an evident peak near E_V_ − 4.6 eV (E_V_ = 0 eV) (Fig. 4e). Notably, the *s, p* orbitals from LiTFSI and Zn(CF_3_SO_3_)_2_ have dominant contributions near both valence band maximum (VBM) and conduction band minimum (CBM), which are consistent with the electronic distributions. Meanwhile, it is noticed that the *s, p* orbitals of H_2_O have considerable overlapping with both LiTFSI and Zn(CF_3_SO_3_)_2_, supporting the formation of the solvation structures. For such an electrolyte, the relatively large bandgap (3.0 eV) between VBM and CBM limits the overall electrical conductivity. As acetonitrile is introduced in the electrolyte (0.5 m + 1 m-H_2_O/AN), a slight downshifting of Zn-3*d* orbitals due to the interactions with acetonitrile (Fig. 4f). More importantly, the *s, p* orbitals of acetonitrile not only dominate the CBM and VBM but also lead to the formation of gap states, which significantly alleviates the energy barrier for electron transfer and improves the electrochemical performances. The orbital overlapping between acetonitrile-*s, p*, and Zn-3*d* is improved while the overlapping between H_2_O-*s, p*, and Zn-3*d* is decreased. Compared with the results of 0.5 m + 1 m-H_2_O electrolyte, the interactions between H_2_O with Zn(CF_3_SO_3_)_2_ and LiTFSI are drastically weakened in the 0.5 m + 1 m-H_2_O/AN electrolyte, demonstrating the change of solvation structure due to the interruption by acetonitrile molecules. On the other hand, as the concentration of LiTFSI increases, a significant downshift of Zn-3*d* orbitals is observed, revealing a further suppression of the interactions between water molecules with both Zn^2+^ cations and acetonitrile molecules (Fig. 4g). Meanwhile, the H_2_O-*s, p* orbitals, instead of the acetonitrile*-s, p* orbitals, become dominant near the Fermi level and the barrier for electron transfer is increased compared to that of 0.5 m + 1 m-H_2_O/AN electrolyte. With the further increase of LiTFSI concentrations, though Zn-3*d* orbitals are upshifted to E_V_ − 5.0 eV, the energy gap between CBM and VBM is enlarged to 4.0 eV, resulting in an even higher barrier for electron transfer (Fig. 4h). In such a circumstance, the interactions between Zn(CF_3_SO_3_)_2_ and LiTFSI are enhanced. However, both Zn(CF_3_SO_3_)_2_ and LiTFSI show weakened interactions with H_2_O and acetonitrile, leading to poor electrochemical performances.

The radial distribution functions (RDFs) of different electrolytes from the MD simulations are also compared. In the 0.5 m + 1 m-H_2_O electrolyte, the main bonding contributions come from the O–H bonds in water molecules and the corresponding hydrogen bonds between water molecules also show an important contribution with the second peak within 1.5–1.6 Å (Fig. 4i). We notice a broad peak after 2.6 Å, which corresponds to the coordination shell formed between Zn ions and water molecules. In contrast, in the water/acetonitrile electrolyte, the peak of water molecules almost disappears, where the stronger peak at 1.1 Å is ascribed to C-H, C≡N bonding of the acetonitrile (Fig. 4j). Compared to the 0.5 m + 1 m-H_2_O electrolyte, the hydrogen bonding network of water is strongly perturbed with no evident peak presented. The weak hydrogen bond and interactions between acetonitrile molecules lead to a peak at 1.8 Å. The coordination shell between Zn ions and acetonitrile molecules is formed at the distance of 2.1 Å, which is much smaller than that with water, indicating the modulations of solvation structure. Meanwhile, the interactions between Zn and water molecules are strongly suppressed, which potentially lowers the possibility during HER. Figure 4k depicts the interaction energies between ions and solvent molecules in different electrolytes. As compared with 0.5 m + 1 m-H_2_O, the interaction energies in the 0.5 m + 1 m-H_2_O/AN electrolyte are reduced, suggesting stronger interactions. As the concentration of LiTFSI increases, the interaction energies increase accordingly due to the weakened interactions, which is consistent with the NMR results. In addition, the mean square displacement (MSD) demonstrates the highest diffusion is induced by the acetonitrile in the 0.5 m + 1 m-H_2_O/AN electrolyte (Fig. 4l). The stronger ion interactions in water/acetonitrile electrolyte, together with higher migration of ions and molecules than those in pristine aqueous electrolyte, not only promote the conductivity of electrolyte but also improve the operation voltage of the supercapacitor.

## Conclusions

In summary, a novel dilute water/acetonitrile electrolyte is developed for the Zn-ion hybrid supercapacitors with superior electrochemical performance. Theoretical calculations verify that acetonitrile molecules have strong interaction with Zn cations, and greatly modulate the solvation structures formed by water molecules in the electrolyte, which is beneficial to expand its electrochemical window at low salt concentrations. The coordination shell formed by acetonitrile not only suppresses Zn corrosion and HER but also promotes overall electrochemical performances. As a result, a high average Coulombic efficiency (97.3% for 3,000 cycles) of repetitive Zn plating/striping is recorded in the dilute 0.5 m + 1 m-H_2_O/AN electrolyte, which also enables an ultra-long lifespan (> 120,000 cycles) in the Zn-ion hybrid supercapacitors. Most importantly, a high operation voltage up to 2.2 V is achieved for the first time in the prototypes of Zn-ion hybrid supercapacitors, ranking the top performances when compared to previous reports (Tables S1 and S2). Moreover, benefiting from the low salt concentrations of the aqueous-aprotic electrolyte, the Zn-ion hybrid supercapacitors can also operate robustly even under harsh conditions in low temperatures to − 30 °C, guaranteeing their applications in broad scenarios. This work provides a new approach to develop novel electrolytes by regulating solvation structures towards Zn-ion hybrid supercapacitors with high energy density and long lifespan.

## Supplementary Information

Below is the link to the electronic supplementary material.

Supplementary file1 (PNG 119 KB)

Supplementary file2 (PNG 107 KB)

Supplementary file3 (PNG 488 KB)

Supplementary file4 (PNG 507 KB)

Supplementary file5 (PNG 170 KB)

Supplementary file6 (PNG 161 KB)

Supplementary file7 (PNG 868 KB)

Supplementary file8 (PNG 238 KB)

Supplementary file9 (PNG 328 KB)

Supplementary file10 (PNG 188 KB)

Supplementary file11 (PNG 524 KB)

Supplementary file12 (PNG 232 KB)

Supplementary file13 (PNG 750 KB)

Supplementary file14 (PNG 1284 KB)

Supplementary file15 (PNG 1104 KB)

Supplementary file16 (PNG 133 KB)

Supplementary file17 (PNG 1280 KB)

Supplementary file18 (PNG 516 KB)

Supplementary file19 (PNG 180 KB)

Supplementary file20 (PNG 2786 KB)

Supplementary file21 (PNG 142 KB)

Supplementary file22 (PNG 67 KB)

Supplementary file23 (PNG 59 KB)

Supplementary file24 (PNG 16 KB)

Supplementary file25 (PNG 89 KB)

Supplementary file26 (DOCX 29 KB)
