# Supplementary figures and images for: Dilute Aqueous-Aprotic Electrolyte Towards Robust Zn-Ion Hybrid Supercapacitor with High Operation Voltage and Long Lifespan
Source: Nanomicro Lett. 2024 Mar 25;16:161. doi: 10.1007/s40820-024-01372-x (PMC10963695; doi:10.1007/s40820-024-01372-x)

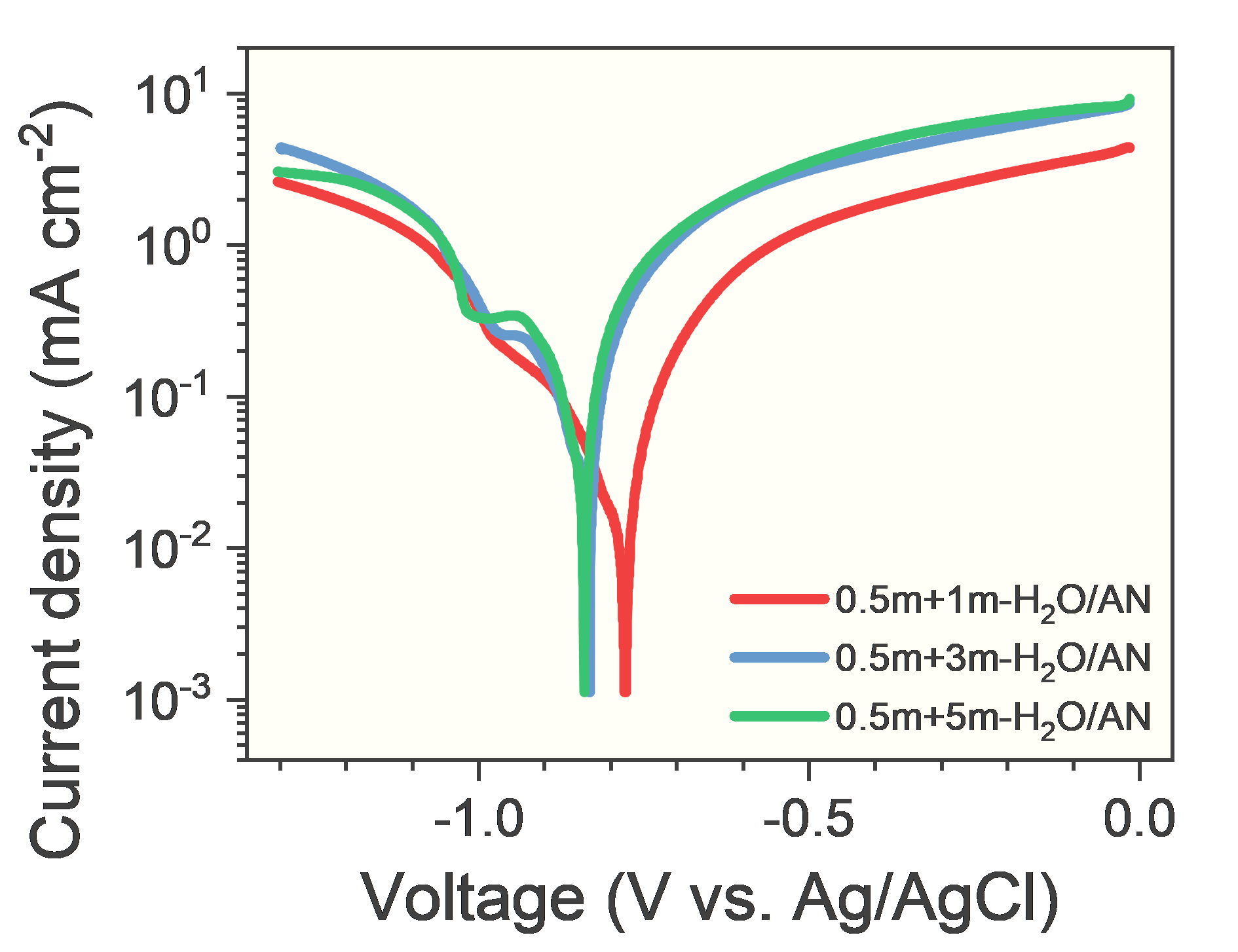

Supplement: Supplementary file 1 — Supplementary file1 (PNG 119 KB) [file 40820_2024_1372_MOESM1_ESM.png]

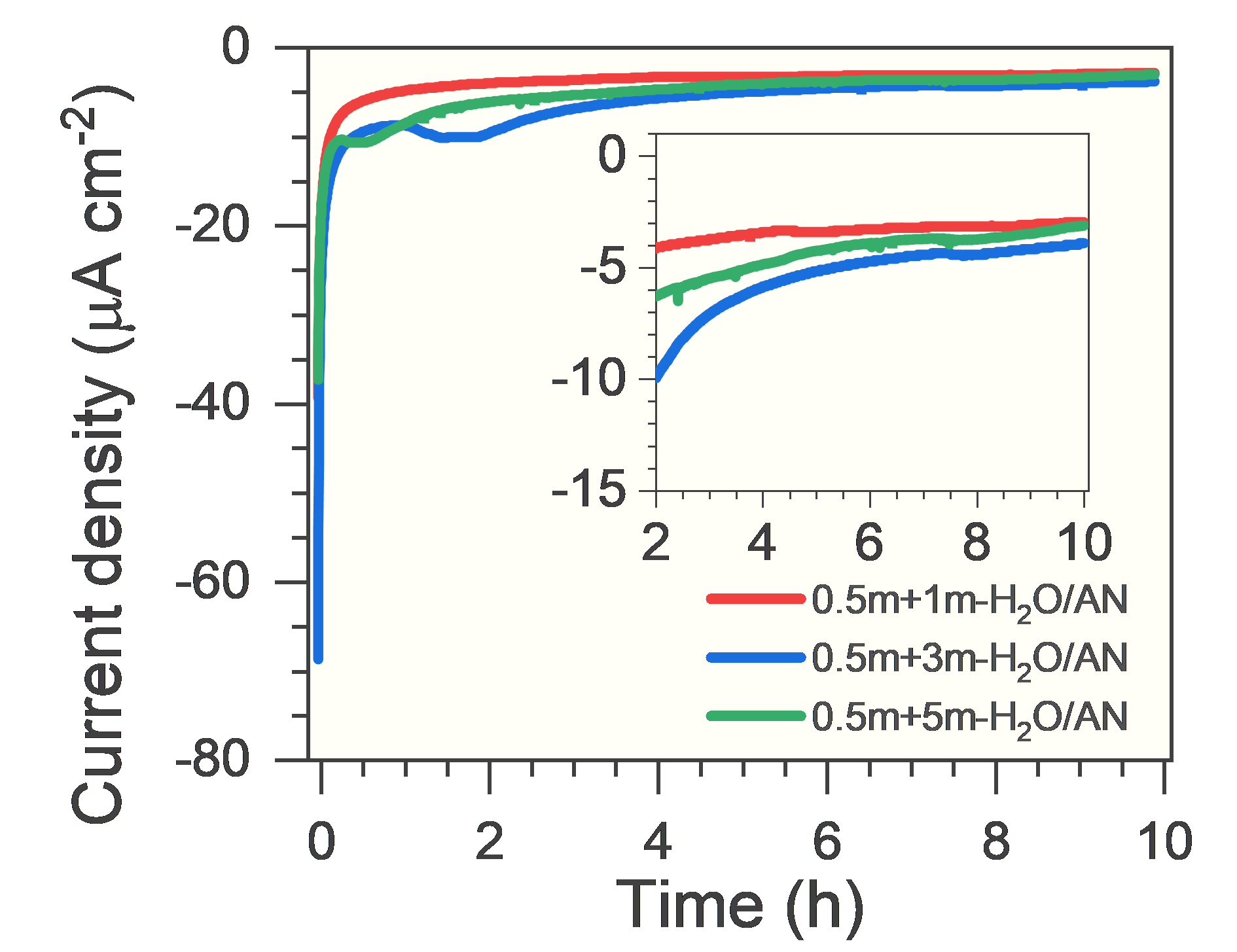

Supplement: Supplementary file 2 — Supplementary file2 (PNG 107 KB) [file 40820_2024_1372_MOESM2_ESM.png]

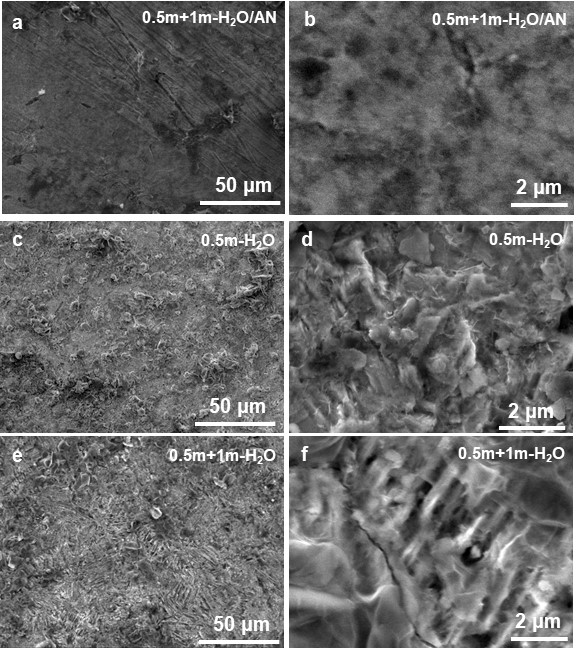

Supplement: Supplementary file 3 — Supplementary file3 (PNG 488 KB) [file 40820_2024_1372_MOESM3_ESM.png]

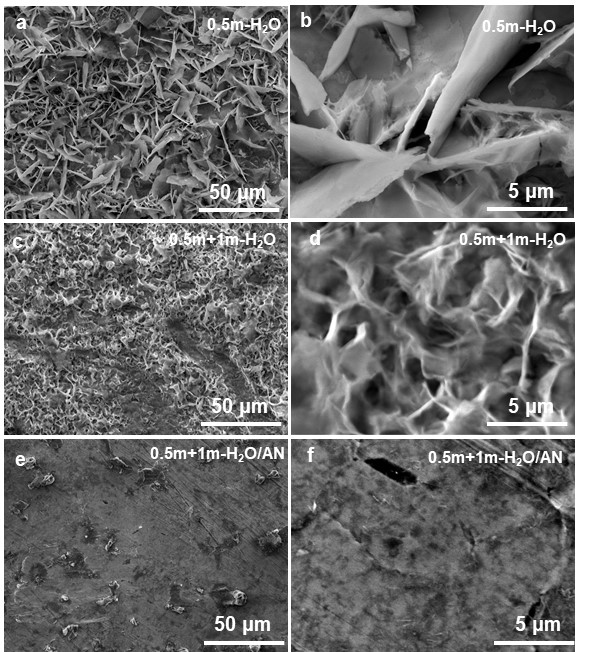

Supplement: Supplementary file 4 — Supplementary file4 (PNG 507 KB) [file 40820_2024_1372_MOESM4_ESM.png]

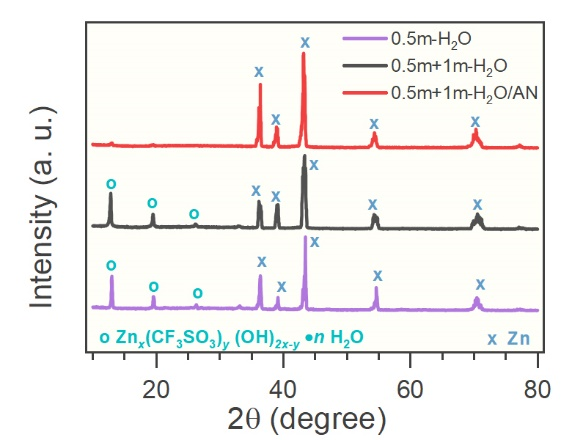

Supplement: Supplementary file 5 — Supplementary file5 (PNG 170 KB) [file 40820_2024_1372_MOESM5_ESM.png]

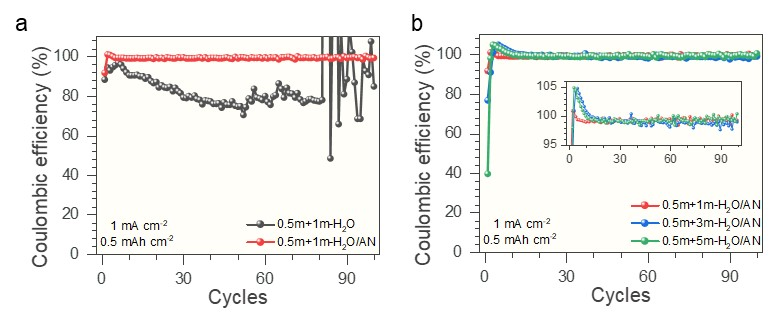

Supplement: Supplementary file 6 — Supplementary file6 (PNG 161 KB) [file 40820_2024_1372_MOESM6_ESM.png]

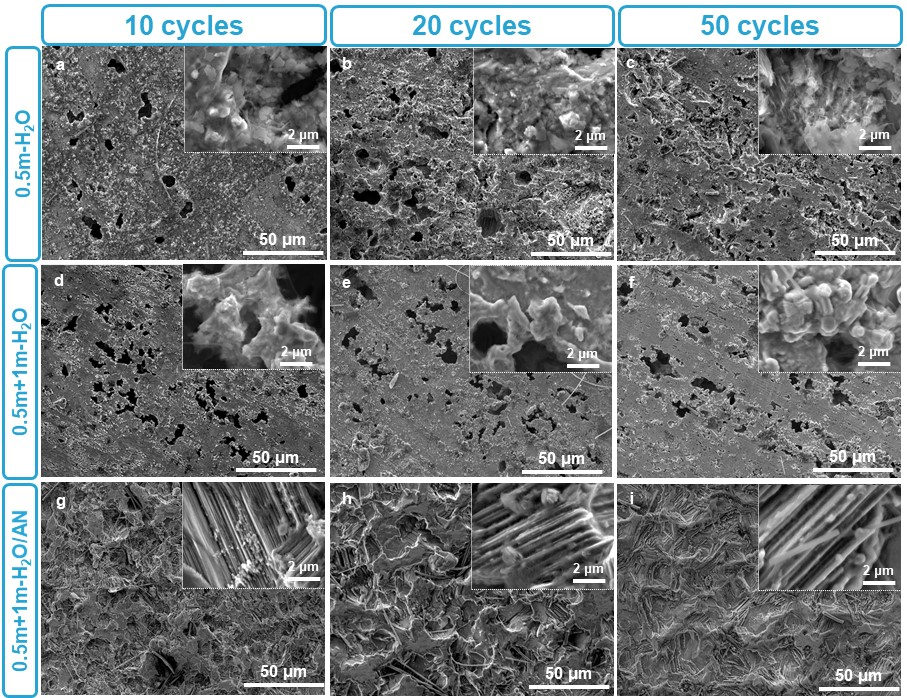

Supplement: Supplementary file 7 — Supplementary file7 (PNG 868 KB) [file 40820_2024_1372_MOESM7_ESM.png]

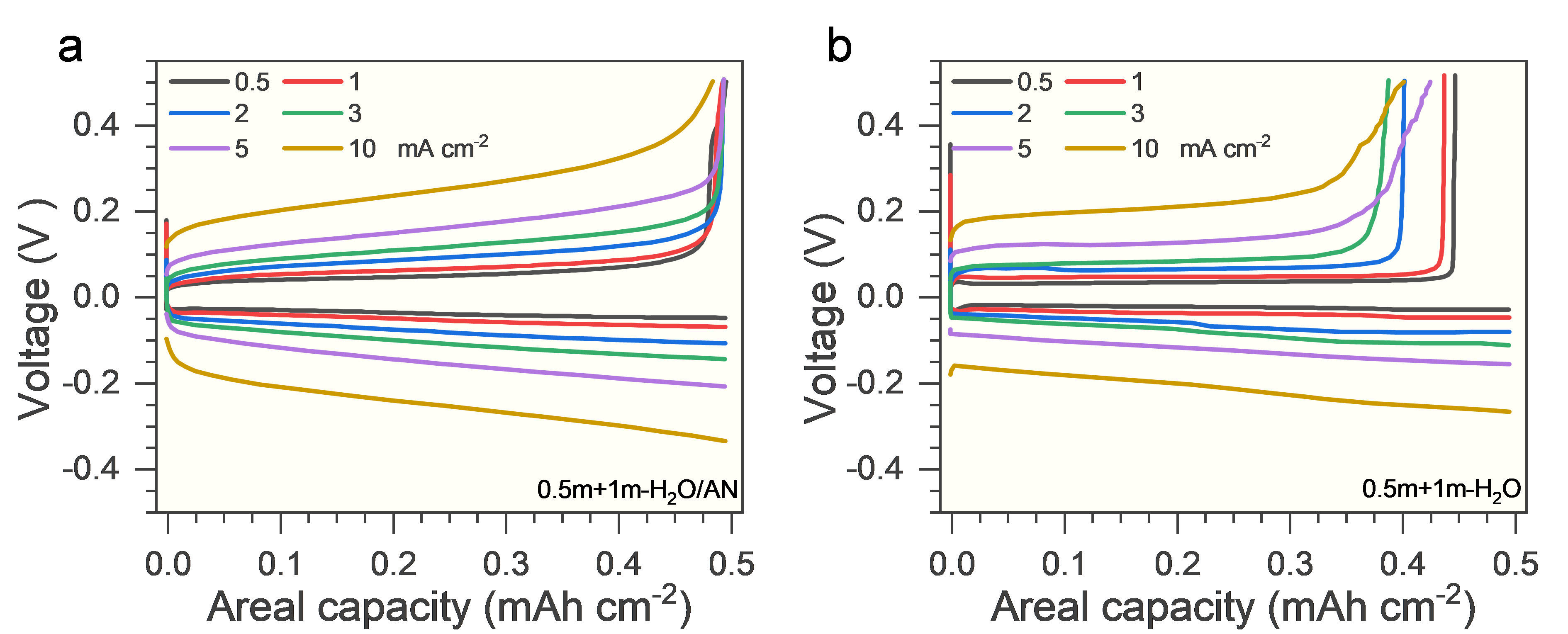

Supplement: Supplementary file 8 — Supplementary file8 (PNG 238 KB) [file 40820_2024_1372_MOESM8_ESM.png]

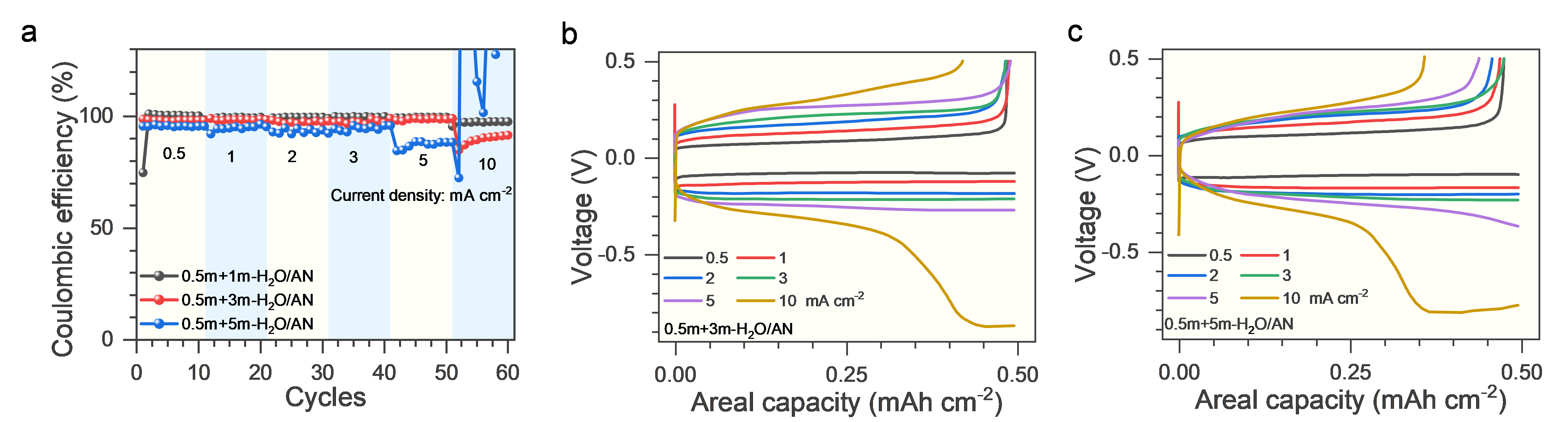

Supplement: Supplementary file 9 — Supplementary file9 (PNG 328 KB) [file 40820_2024_1372_MOESM9_ESM.png]

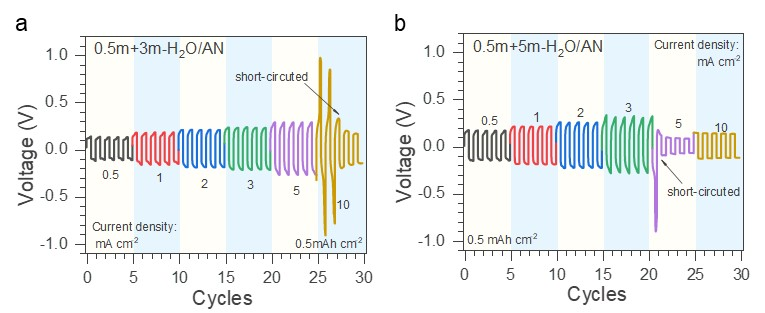

Supplement: Supplementary file 10 — Supplementary file10 (PNG 188 KB) [file 40820_2024_1372_MOESM10_ESM.png]

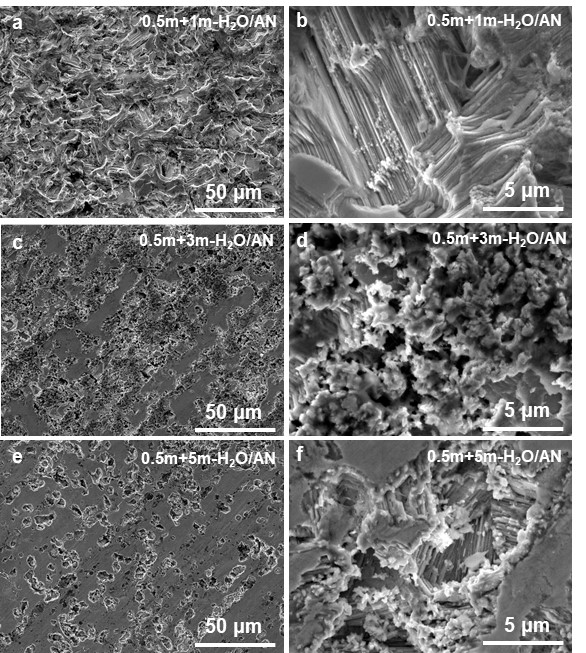

Supplement: Supplementary file 11 — Supplementary file11 (PNG 524 KB) [file 40820_2024_1372_MOESM11_ESM.png]

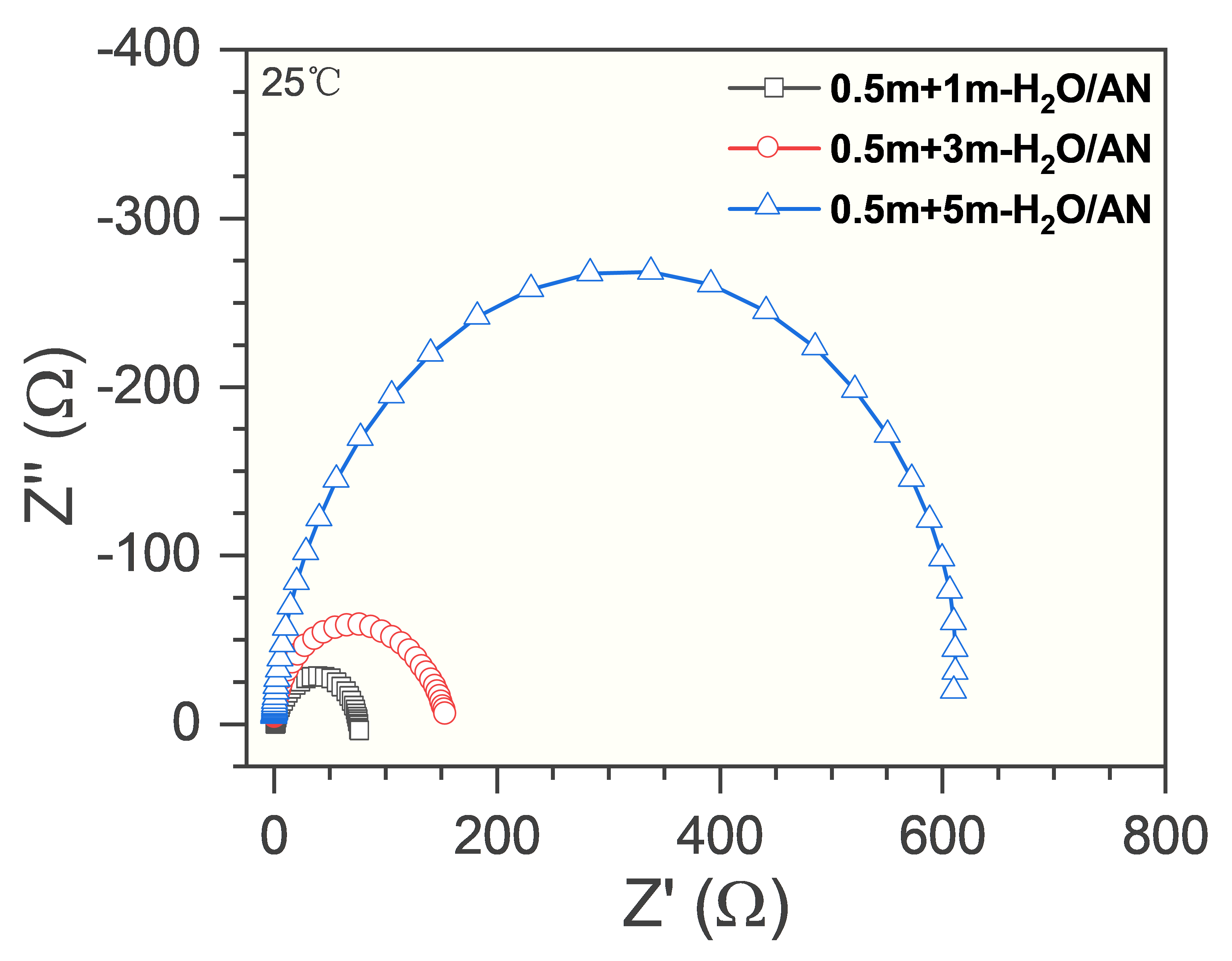

Supplement: Supplementary file 12 — Supplementary file12 (PNG 232 KB) [file 40820_2024_1372_MOESM12_ESM.png]

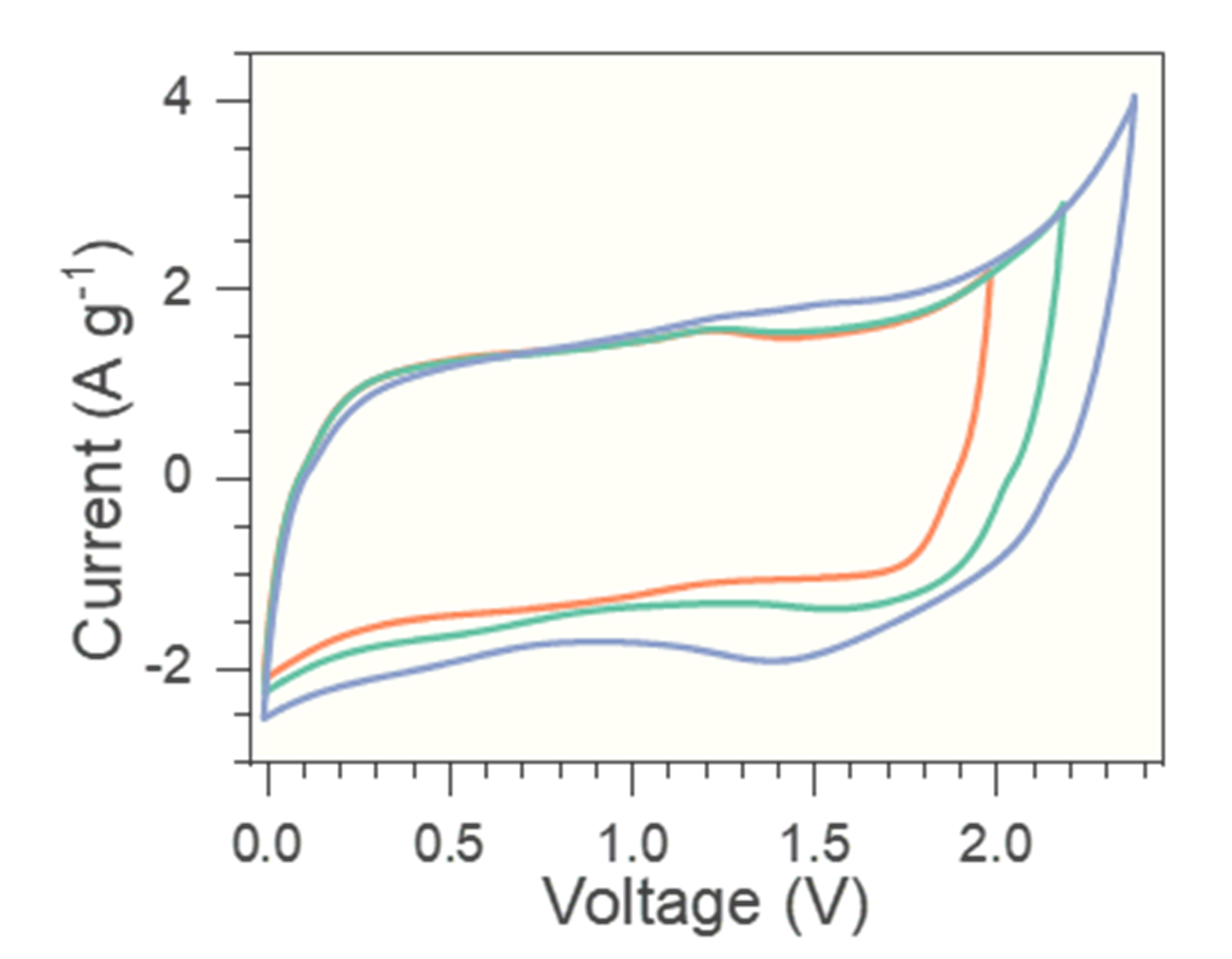

Supplement: Supplementary file 13 — Supplementary file13 (PNG 750 KB) [file 40820_2024_1372_MOESM13_ESM.png]

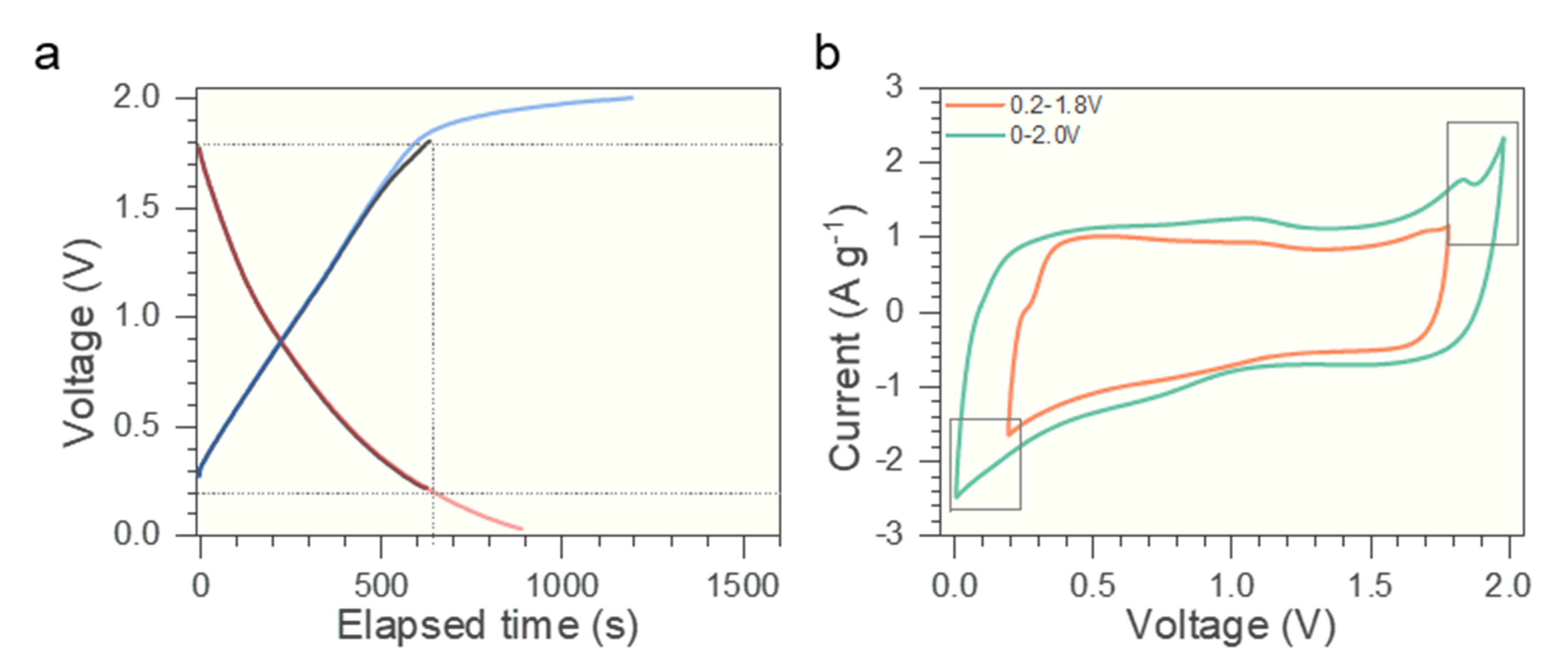

Supplement: Supplementary file 14 — Supplementary file14 (PNG 1284 KB) [file 40820_2024_1372_MOESM14_ESM.png]

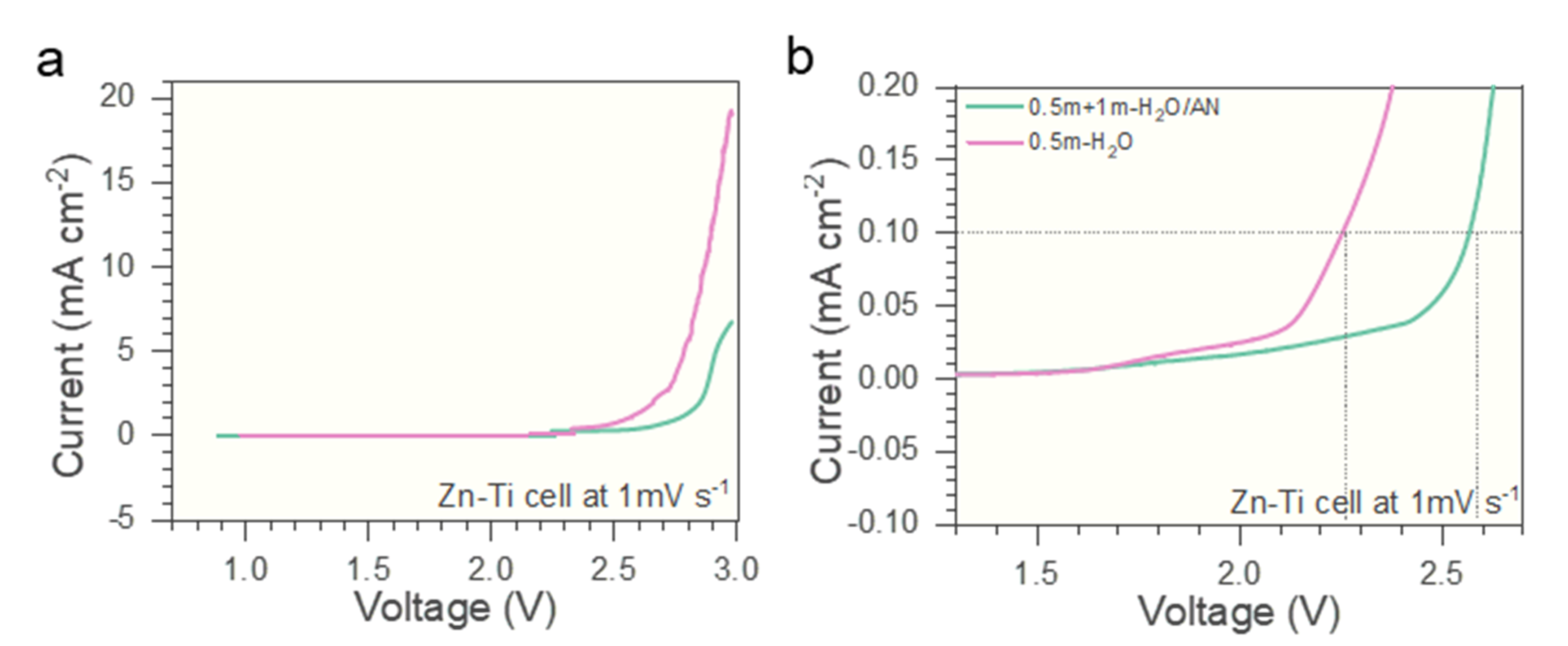

Supplement: Supplementary file 15 — Supplementary file15 (PNG 1104 KB) [file 40820_2024_1372_MOESM15_ESM.png]

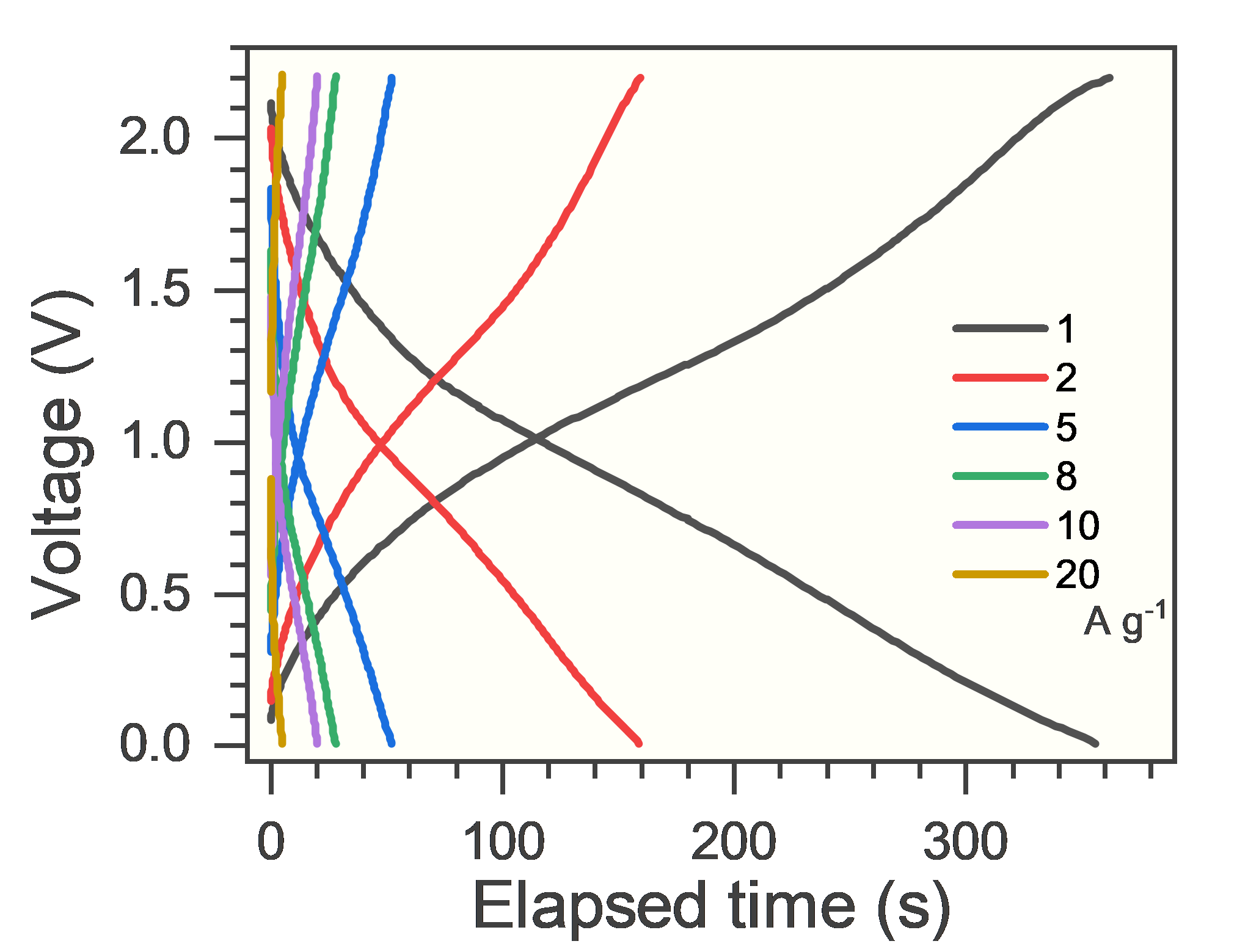

Supplement: Supplementary file 16 — Supplementary file16 (PNG 133 KB) [file 40820_2024_1372_MOESM16_ESM.png]

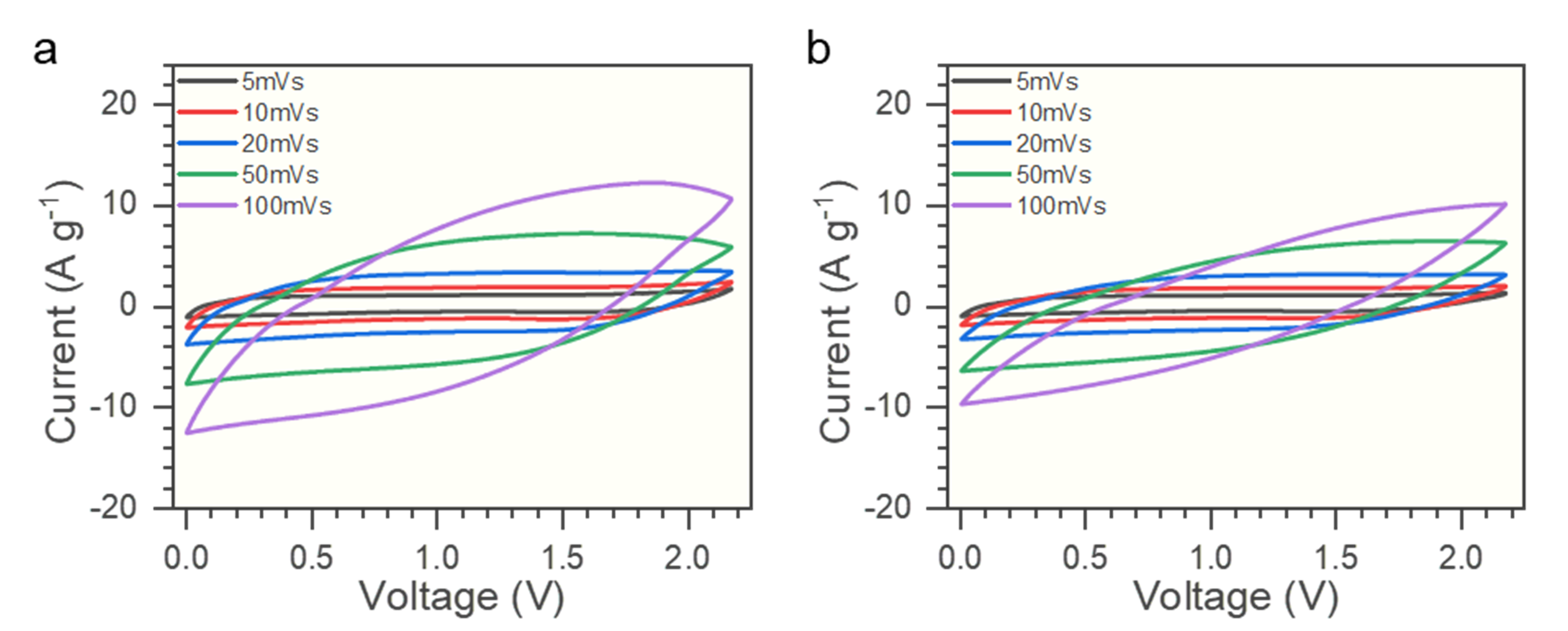

Supplement: Supplementary file 17 — Supplementary file17 (PNG 1280 KB) [file 40820_2024_1372_MOESM17_ESM.png]

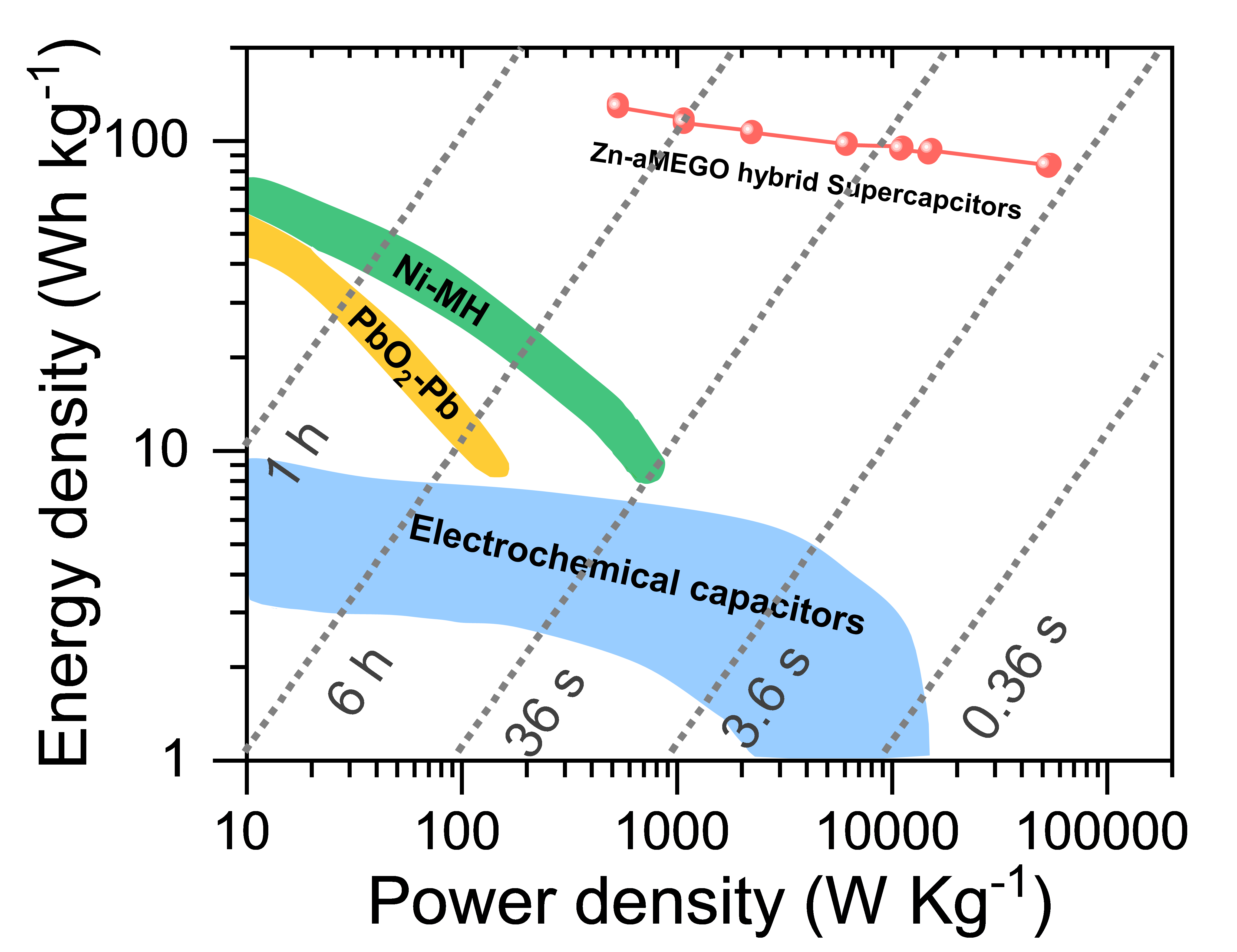

Supplement: Supplementary file 18 — Supplementary file18 (PNG 516 KB) [file 40820_2024_1372_MOESM18_ESM.png]

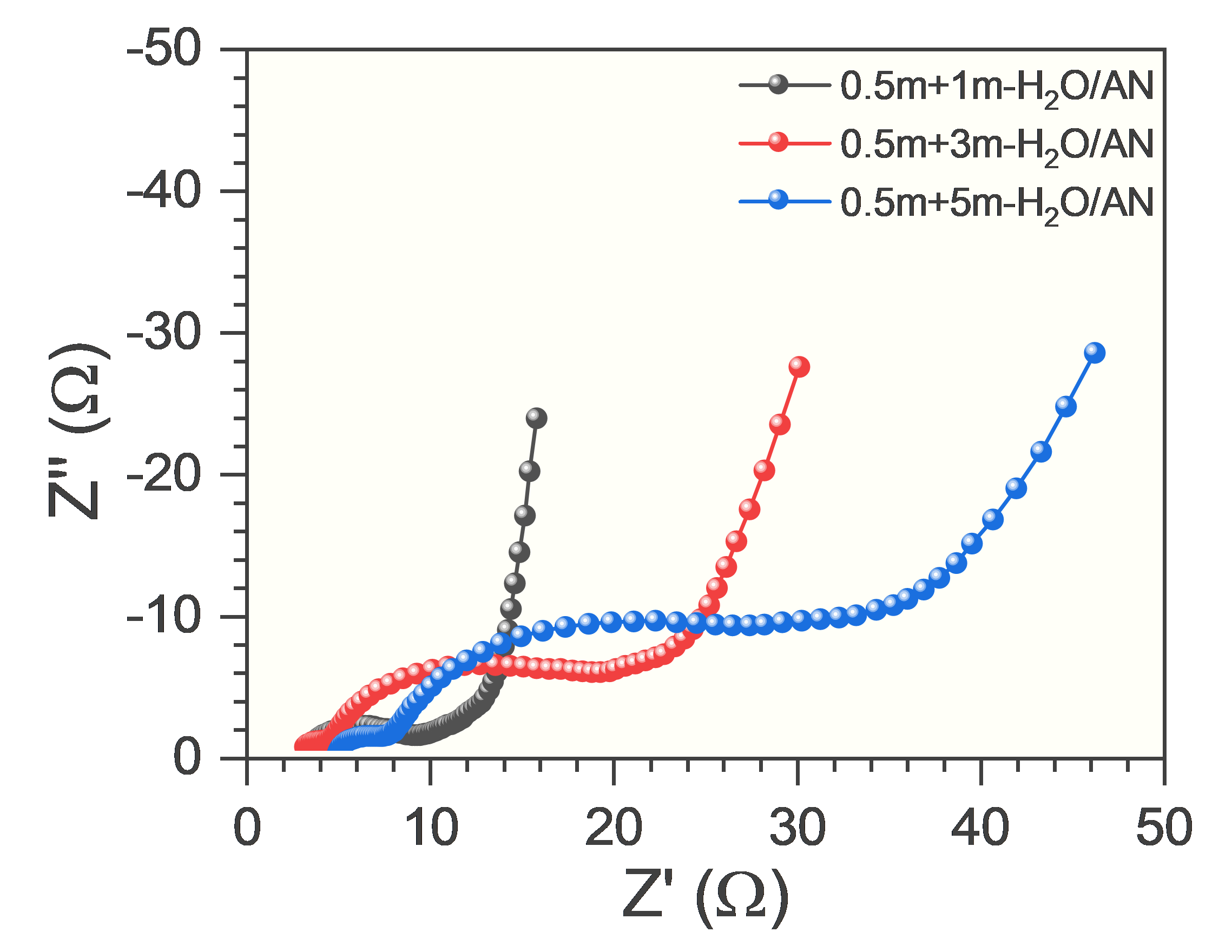

Supplement: Supplementary file 19 — Supplementary file19 (PNG 180 KB) [file 40820_2024_1372_MOESM19_ESM.png]

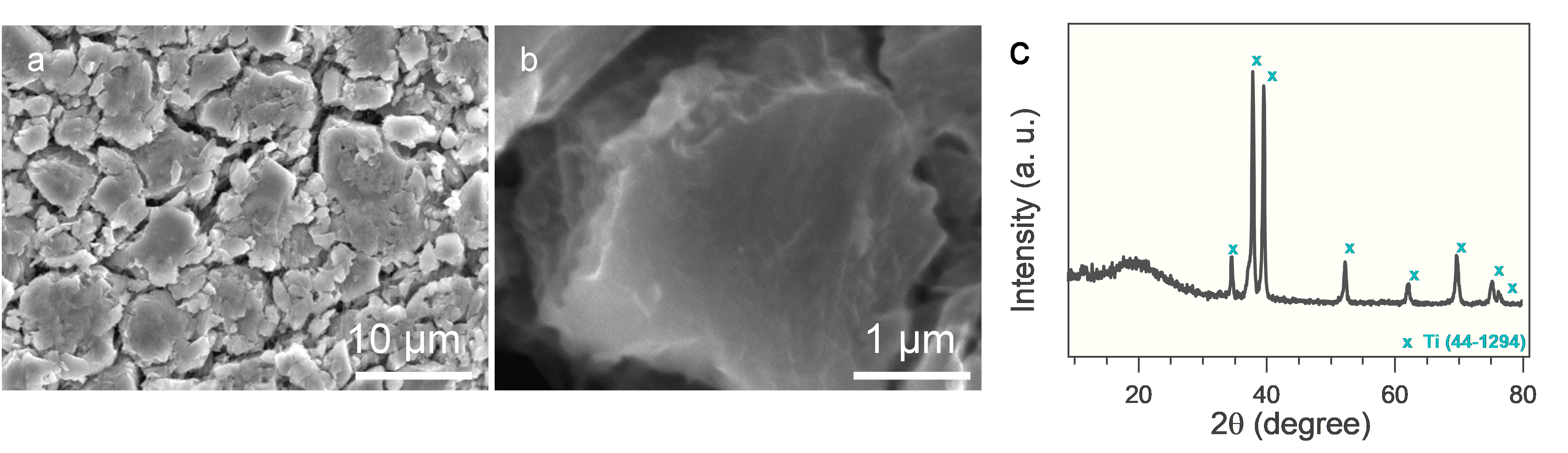

Supplement: Supplementary file 20 — Supplementary file20 (PNG 2786 KB) [file 40820_2024_1372_MOESM20_ESM.png]

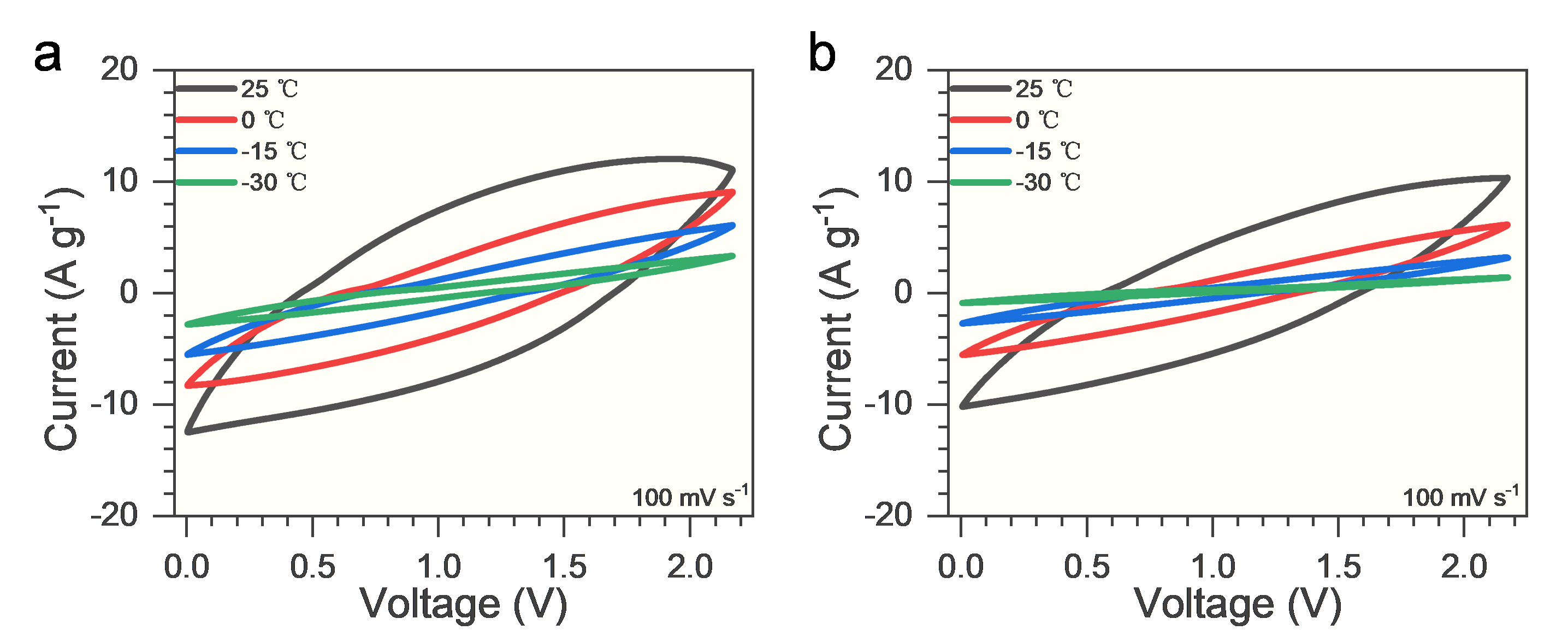

Supplement: Supplementary file 21 — Supplementary file21 (PNG 142 KB) [file 40820_2024_1372_MOESM21_ESM.png]

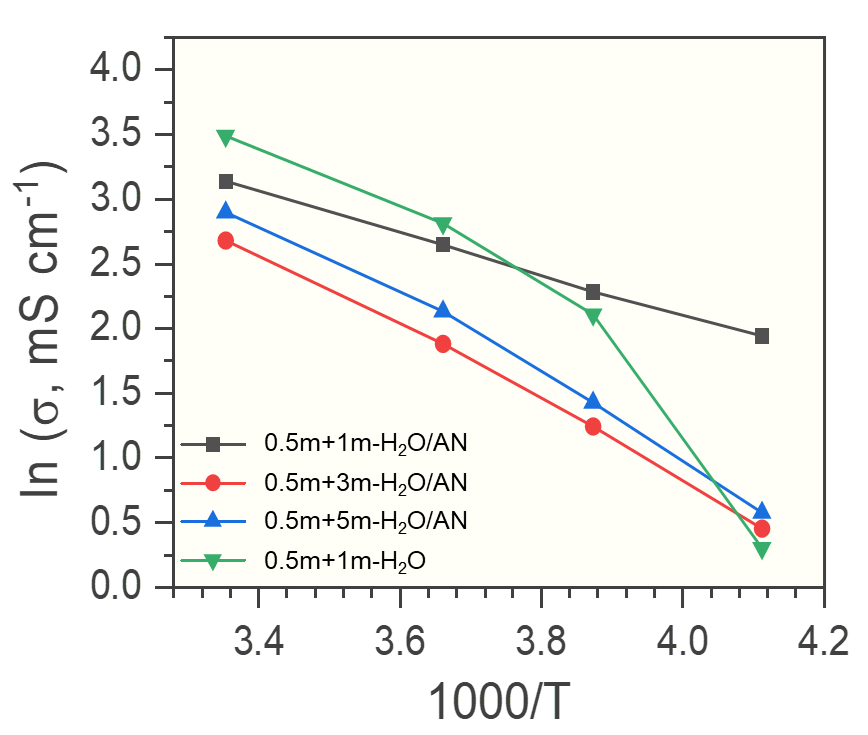

Supplement: Supplementary file 22 — Supplementary file22 (PNG 67 KB) [file 40820_2024_1372_MOESM22_ESM.png]

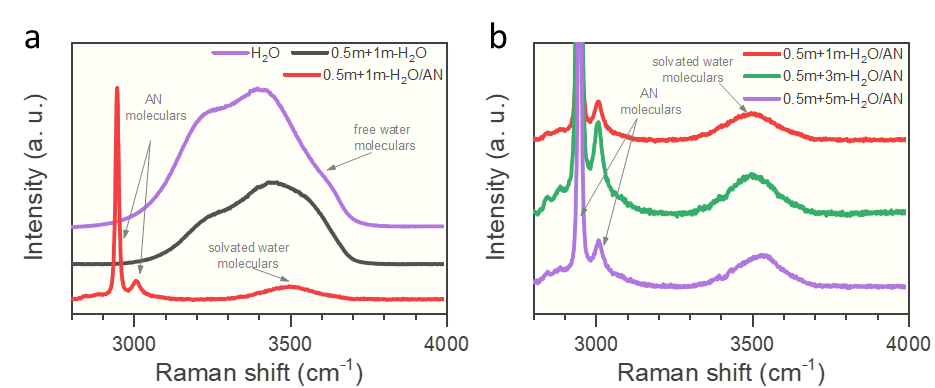

Supplement: Supplementary file 23 — Supplementary file23 (PNG 59 KB) [file 40820_2024_1372_MOESM23_ESM.png]

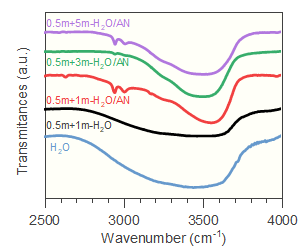

Supplement: Supplementary file 24 — Supplementary file24 (PNG 16 KB) [file 40820_2024_1372_MOESM24_ESM.png]

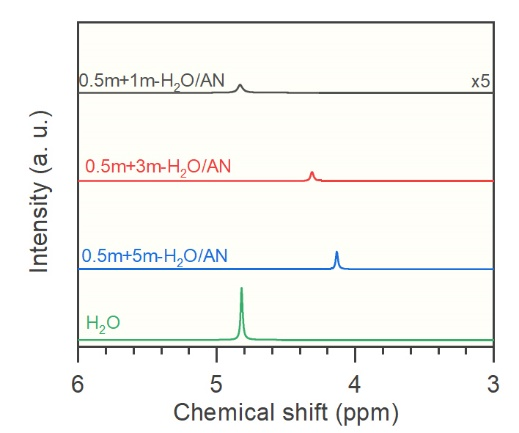

Supplement: Supplementary file 25 — Supplementary file25 (PNG 89 KB) [file 40820_2024_1372_MOESM25_ESM.png]
