# Supplementary material for: Dilute Aqueous-Aprotic Electrolyte Towards Robust Zn-Ion Hybrid Supercapacitor with High Operation Voltage and Long Lifespan
Source: Nanomicro Lett. 2024 Mar 25;16:161. doi: 10.1007/s40820-024-01372-x (PMC10963695; doi:10.1007/s40820-024-01372-x)
Supplement: Supplementary file 26 — Supplementary file26 (DOCX 29 KB) [file 40820_2024_1372_MOESM26_ESM.docx]

**Table S1. Performance comparison with Zn-ion hybrid supercapacitors**

| **Energy storage system**  **(Cathode//Electrolyte//Anode)** | **Operating**  **voltage** | **Capacitance** | **Energy density** | **Cycling stability** | **Reference** |
| --- | --- | --- | --- | --- | --- |
| AC//2 M ZnSO_4_ (aq.)//Zn | 0.2~1.8 V | 272.25 F/g (0.1 A/g) | 84.0 Wh/Kg | 91% after  10, 000 cycles | *Energy Storage Mater.*  2018,13, 96–102 |
| AC//1 M Zn(CF_3_SO_3_)_2_ (DOL-DME)//Zn | 0~1.8 V | 116 F/g  (0.5 A/g) | 44.9 Wh/Kg | \ | *Energy Storage Mater.*  2018,13, 96–102 |
| AC//1M Zn(CF_3_SO_3_)_2_ (AN)//Zn | 0~1.8 V | 130 F/g  (2 A/g) | 52.7 Wh/Kg | 92% after  22, 000 cycles | *Energy Storage Mater.*  2018,13, 96–102 |
| AC//2M ZnSO_4_ (aq.)//Zn | 0.5~1.5 V | 259 F/g  (0.05 A/g) | 36.0 Wh/kg | 100% after  10, 000 cycles | *Adv. Mater.*  2019, 31, 1806005 |
| CNT// ZnSO_4_ (aq. or gel.)//Zn | 0.2~1.8 V | 83.2 mF/cm^2^  (1 mA/cm^2^) | 29.6 μWh/cm^2^ | 87% after  6, 000 cycles | *Energy Environ. Sci.*,  2018, 11, 3367--3374 |
| N-PC//1M ZnSO_4_//Zn | 0-1.8V | 177.8 mAh g^-1^  (4.2 A g^-1^) | 107.3 Wh kg^-1^ | 73.6% after  100,000 cycles | *Adv. Mater.,*  *2019, 1904948* |
| L-NS-CNS//2 M ZnSO_4_//Zn | 0.2-1.8 V | 233.4 F g^-1^  (0.1 A g^-1^) | 91 Wh kg^-1^ | 94.2% after  18, 000 cycles | *Adv. Funct. Mater. 2022, 32, 2209914* |
| AC//ZnMg-0.1//Zn | 0.2-1.85 V | 154 mAh g^-1^  (1 A g^-1^) | \ | 98.7% after  10, 000 cycles | *Adv. Energy Mater., 2021, 2101158* |
| O-PC//3M Zn(ClO_4_)_2_//Zn | 0-1.9 V | 340.7 F g^-1^  (0.1 A g^-1^) | 104.8 Wh kg^-1^ | 99.2% after  30, 000 cycles | *Adv. Energy Mater.2020, 10, 2001705* |
| **aMEGO//0.5m+1m-H_2_O/AN electrolyte//Zn** | **0~2.2 V** | **192 F/g**  **(0.5 A/g)** | **129 Wh kg^-1^** | **88% after**  **120, 000 cycles** | ***This work*** |

**Table S2. . Coulombic efficiency comparison of different electrolyte systems**

| **Electrolyte system** | **Test Protocol** | **CE (%)** | **Ionic conductivity** | **Reference** |
| --- | --- | --- | --- | --- |
| 4m Zn(OTF)_2_+0.5m Me_3_EtNOTF-H_2_O | 0.5 mA cm^-2^, 0.5 mAh cm^-2^ | 99.9%  (1000 cycles) | ~25 mS cm^-1^ | *Nat. Nanotech., 2021, 16, 902* |
| 1m Zn(TFSI)_2_+20m LiTFSI-H_2_O | CV at 1 mV s^-1^  -0.6-0.6 V | 99.7%  (200 cycles) | \ | *Nat. Mater. 2018, 17, 543* |
| 4m Zn(BF_4_)_2_-EG | 1 mA cm^-2^,  0.5 mAh cm^-2^ | 99.4%  (400 cycles) | 4.5 mS cm^-1^ | *Nat. Sustain., 2022, 5, 205* |
| Zn(CF_3_SO_3_)_2_-Methanol | 2.93 mA cm^-2^  2.93 mAh cm^-2^ | 99.9%  (140 cycles) | 8-15 mS cm^-1^ | *Proc. Natl. Acad. Sci. U. S. A., 2022, 24, e2121138119* |
| Zn(ClO_4_)_2_-H_2_O- Succinonitrile eutectic electrolyte | 0.5 mA cm^-2^,  0.5 mAh cm^-2^ | 98.4%  (45-90 cycles) | 17.66 mS cm^-1^ | *Joule, 2020, 7, 1557* |
| ZnSO_4_-H_2_O-Methanol | 1 mA cm^-2^,  0.5 mAh cm^-2^ | 99.7%  (900 cycles) | 1.68 mS cm^-1^ | *Angew Chem Int Edit 2021, 60 (13), 7366* |
| ZnSO_4_-glucose-H_2_O | 1 mA cm^-2^,  0.5 mAh cm^-2^ | 97.2%  (200 cycles) | \ | *Angew Chem Int Edit 2021, 60 (33), 18247* |
| **0.5m Zn(CF_3_SO_3_)_2_+1m LiTFSI-H_2_O/AN** | **5 mA cm^-2^,**  **0.5 mAh cm^-2^** | **97.3%**  **(3000 cycles)** | **23 mS cm^-1^** | ***This work*** |
